# Low-dose lysergic acid diethylamide alters subjective visual perception but not visual contrast surround suppression

**DOI:** 10.1093/nc/niag050

**Published:** 2026-08-20

**Authors:** Lucca F Jaeckel, Deborah Logvinski, Mika Kanana, Felix Müller, Nicolai Rohner, Anna M Becker, Matthias E Liechti, Philipp Sterzer

**Affiliations:** Division of Translational Psychiatry, Department of Psychiatry (UPK) and Department of Clinical Research, University of Basel, Wilhelm Klein-Strasse 27, 4002 Basel, Switzerland; Division of Translational Psychiatry, Department of Psychiatry (UPK) and Department of Clinical Research, University of Basel, Wilhelm Klein-Strasse 27, 4002 Basel, Switzerland; Division of Translational Psychiatry, Department of Psychiatry (UPK) and Department of Clinical Research, University of Basel, Wilhelm Klein-Strasse 27, 4002 Basel, Switzerland; Clinical Research Unit for Substance-Based Therapy, Department of Psychiatry (UPK), University of Basel, Wilhelm Klein-Strasse 27, 4002 Basel, Switzerland; Division of Clinical Pharmacology and Toxicology, Department of Biomedicine and Department of Clinical Research, University Hospital Basel and University of Basel, Schanzenstrasse 55, 4031 Basel, Switzerland; Division of Translational Psychiatry, Department of Psychiatry (UPK) and Department of Clinical Research, University of Basel, Wilhelm Klein-Strasse 27, 4002 Basel, Switzerland; Clinical Research Unit for Substance-Based Therapy, Department of Psychiatry (UPK), University of Basel, Wilhelm Klein-Strasse 27, 4002 Basel, Switzerland; Division of Clinical Pharmacology and Toxicology, Department of Biomedicine and Department of Clinical Research, University Hospital Basel and University of Basel, Schanzenstrasse 55, 4031 Basel, Switzerland; Division of Clinical Pharmacology and Toxicology, Department of Biomedicine and Department of Clinical Research, University Hospital Basel and University of Basel, Schanzenstrasse 55, 4031 Basel, Switzerland; Division of Translational Psychiatry, Department of Psychiatry (UPK) and Department of Clinical Research, University of Basel, Wilhelm Klein-Strasse 27, 4002 Basel, Switzerland

**Keywords:** states of consciousness, psychophysics, pharmacology, perception

## Abstract

Theoretical models and empirical evidence suggest that psychedelics alter contextual influences on perception. To test this, we examined contrast surround suppression, a visual illusion in which a grating appears lower in contrast when embedded in a high-contrast surround. While psilocybin has been reported to enhance this and related illusions at moderate-to-high doses, it is unknown whether low-dose Lysergic Acid Diethylamide (LSD) produces similar effects. In a randomized, double-blind, within-subjects crossover study, N = 30 healthy participants received placebo, 10, and 20 μg of LSD (base-equivalent). In each session, participants performed a contrast discrimination task and rated subjective drug effects. LSD did not measurably influence contrast surround suppression. However, participants reported subjective visual changes, including blurrier, more saturated, and more dynamical visual perception. These subjective alterations did not correlate with individual differences in contrast-surround suppression. Our findings indicate a dissociation between subjective experience and early visual contextual processing, suggesting that subjective visual effects of low-dose LSD may arise through mechanisms distinct from those mediating contrast surround suppression. Future research should test whether higher doses of LSD affect contrast surround suppression.

## Introduction

Classical psychedelics like Lysergic Acid Diethylamide (LSD) and psilocybin induce a markedly altered state of consciousness through agonism at the 5-hydroxytryptamine 2A (5-HT2A) receptor (Nichols 2016, Liechti 2017). Studying this state provides insights for translational psychiatric and basic consciousness research. Psychiatrically, the pharmacologically altered state of consciousness provides a laboratory model of hallucinatory experience, and specific phenomenological features of this state seem to predict therapeutic effects of psychedelics in treating psychiatric conditions (Vollenweider et al. 1998, Hermle and Kraehenmann 2016, Aday et al. 2020, Yaden and Griffiths 2021, Aqil and Roseman 2022). In terms of basic consciousness research, the psychedelic drug-induced state permits experimental laboratory research into the dimensions of conscious experience and how they relate to cognitive and neuronal processes (Carter et al. 2005, Bayne and Carter 2018, Yaden et al. 2021). Together, these lines of research highlight psychedelics as a unique tool to investigate 5-HT2A receptor-dependent neuromodulation of large-scale cortical computations and conscious experience.

Visual perception provides a particularly tractable domain for probing these processes experimentally (Heeger et al. 2017), and systematic investigations of perception after psychedelic administration have critically advanced our understanding of such computational alterations (Aqil and Roseman 2022). Psychophysical and neurophysiological studies using psilocybin in humans indicate that psychedelics predominantly affect higher-order perceptual phenomena associated with feedback processing (Carter et al. 2004, Kometer et al. 2011, 2013). Together with resting-state neuroimaging studies, neurobiological insights, and the phenomenology of psychedelics, these findings stimulated the formulation of a theoretical predictive coding model of psychedelic drug effects. This model posits that psychedelics reduce the effects of feedback signaling on perception (Carhart-Harris and Friston 2019). However, animal model research found that 5-HT2A receptor stimulation reduces V1 visually-evoked responses, which may indicate reduced feedforward signaling (Seillier et al. 2017, Michaiel et al. 2019, Azimi et al. 2020). It should be noted, though, that interspecies differences, such as in 5-HT2A receptor distribution (Watakabe et al. 2009), limit direct translation to humans. These and other conflicting findings have motivated competing models of how psychedelics alter feedback and feedforward signaling (Vollenweider and Geyer 2001, Pink-Hashkes et al. 2017, Aqil and Roseman 2022, Doss et al. 2022, Safron et al. 2025). Studying perceptual biases and perceptual decision-making after psychedelic administration provides a powerful approach to disentangle psychedelic effects on distinct levels of the cortical hierarchy.

Contrast surround suppression refers to a visual perceptual bias in which observers underestimate the contrast of a grating when it is embedded in a high-contrast surround (Dakin et al. 2005, Yoon et al. 2010, Schallmo et al. 2015). This bias likely reflects both lateral inhibition within V1 and top-down inhibitory feedback from extrastriate areas. It is consistently reduced in schizophrenia, where it was shown to correlate with lower GABA concentrations in V1 (Dakin et al. 2005, Yoon et al. 2010, Seymour et al. 2013, Tibber et al. 2013, Serrano-Pedraza et al. 2014, Schallmo et al. 2015). However, GABA concentrations have also been shown to inversely correlate with contrast surround suppression in healthy volunteers (Cook et al. 2016), while two other studies, one in healthy volunteers and one in young adults with autism, found no association between GABA and contrast surround suppression at all (Schallmo et al. 2018, 2020). Contrast surround suppression represents a specific psychophysical instance of the broader phenomenon of surround suppression, in which visual cortical neurons’ responses to stimuli in their classical receptive field are attenuated by responses from the extra-classical receptive field (Allman et al. 1985, Carandini and Heeger 2012, Cavanaugh et al. 2002, Phillips et al. 2021). In mice, the 5-HT2A receptor agonist 2,5-Dimethoxy-4-iodoamphetamine (DOI) has been shown to reduce such surround suppression effects at the neural level (Michaiel et al. 2019). Complementing these findings, a recent pilot study in humans reported that a high dose of psilocybin enhanced contrast surround suppression and that this correlated with subjective visual drug effects (Swanson et al. 2024). Similarly, administration of low to moderate doses of psilocybin increased susceptibility to the Ebbinghaus illusion, a size perception bias driven by spatial context. This effect correlated positively with subjective visual alterations and negatively with an fMRI-based, model-derived indicator of surround suppression derived from population receptive field mapping (Aqil et al. 2025). Together, these findings highlight contrast surround suppression as a tractable perceptual paradigm to study how psychedelics modulate cortical computations in humans.

Building on these findings, we investigated whether low doses of LSD affect visual contrast surround suppression in a randomized, controlled, crossover trial in healthy volunteers. Participants received placebo, as well as 10 and 20 μg of LSD (base-equivalent), which can be considered micro- to minidoses (Holze et al. 2021b, Nutt et al. 2025). The lower (“micro”) dose was expected to be indistinguishable from placebo and the higher (“mini”) dose was expected to induce mild subjective and autonomic effects. In each session, participants performed multiple tasks while EEG data were recorded.

Four hours after drug administration, participants performed 100 trials of a two-alternative forced-choice (2AFC) contrast discrimination task to assess contrast surround suppression. We used Visual Analog Scales and the Five-Dimensional Altered States of Consciousness rating scale (5D-ASC; Dittrich et al. 2006) to assess subjective drug effects. The hypotheses and planned analyses for the task data were preregistered on the Open Science Framework prior to unblinding and computation of group statistics, but after fitting and inspecting individual session psychometric curves to confirm data quality (https://osf.io/59amf).

We tested two competing hypotheses regarding LSD’s effect on contrast surround suppression. First, based on Michaiel et al.’s (2019) mouse model findings, we hypothesized that [H1a:] LSD reduces contrast surround suppression. Conversely, based on Swanson et al.’s (2024) findings, we also considered the alternative that [H1b:] LSD enhances contrast surround suppression. Second, we tested whether [H2:] the effect of LSD on reported subjective visual changes correlates with its effect on visual contrast surround suppression. Third, in addition to these preregistered hypotheses, we asked whether [H3:] low-dose LSD induces acute general and visual subjective effects.

## Methods

### Study design

The study used a double-blind, within-subjects crossover design. All participants were administered 0, 10, and 20 μg of LSD (base-equivalent doses), in three separate test sessions with a washout period of ≥7 days. Equal numbers of participants were randomized into each of the six possible dosing sequences by the Good Manufacturing Practice (GMP) facility that produced the formulation to preserve blinding.

Each test session started at 8:30 a.m. and lasted 5 h until 1:30 p.m. Upon arrival, participants received a standardized oat bar breakfast. LSD or placebo was administered orally at 9:00 a.m. Afterwards, the participants performed multiple EEG and behavioral tasks. The contrast surround suppression task was completed 4 h post-administration, following ~2 h of engagement in other visual and EEG paradigms with breaks interspersed throughout.

Prior to the test sessions, participants underwent a screening session during which we assessed their eligibility, obtained baseline trait questionnaires, and introduced them to the cognitive tasks. The study was conducted in accordance with the Declaration of Helsinki and International Conference on Harmonization Guidelines in Good Clinical Practice guidelines. Approval was obtained from the Ethics Committee of Northwest Switzerland (EKNZ; #2023-00763) and Swiss Federal Office for Public Health (BAG). The study was registered at ClinicalTrials.gov (NCT05976698).

### Drug

The 10 and 20 μg doses of LSD (base equivalent) were administered as oral solution (1 ml) of LSD tartrate dissolved in 20% ethanol. The LSD solution and corresponding placebo (20% ethanol without LSD) was produced by Dr. Hysek AG (Biel, Switzerland) in accordance with GMP.

### Study participants

Our target sample size was N = 30 participants (15 female) aged 18–65. Dropouts were replaced to ensure a final sample of 30 complete datasets. The participants whose data were analyzed in the present study were on average 28.1 years old (SD = 8.12; see below for explanations of data in−/exclusion). Inclusion criteria were: BMI 18–29; fluency in German; normal or corrected-to-normal vision; willingness to refrain from operating vehicles or heavy machinery 24 h after dosing; abstinence from illicit substances during the study; alcohol consumption limited to one standard drink the night before study sessions with no alcohol consumption for 24 h after; abstinence from caffeine 12 h before study sessions until their end; use of effective contraception throughout the study; and adequate cognitive task performance during the screening. Exclusion criteria were: recent participation in another clinical trial; (planned) pregnancy or nursing; current use of psychoactive, illicit, or contraindicated drugs; prior psychedelic use exceeding 20 times or use within the past 2 months; smoking more than five cigarettes a day; drinking more than 20 alcoholic standard drinks a week; severe chronic or acute medical conditions; hypertension or hypotension; lifetime history or current major mental health disorder; and first-degree family history of a primary psychotic disorder. Participants were screened for current and lifetime major mental health disorders, such as major depressive disorder, psychotic disorders, or substance abuse disorders as defined by ICD-10 criteria, which required treatment and/or caused significant impairment. Lifetime drug use was assessed through self-report at the screening. Somatic health was assessed using self-report, complemented by measuring body temperature, blood pressure, body weight, and height. Pregnancy tests and urine drug screens were conducted during screening and prior to drug administration in each test session to confirm abstinence and pregnancy status.

Participants were recruited via adverts posted on the university of Basel online marketplace and financially compensated for study participation.

### Subjective effects

We used Visual Analog Scales (VAS; 100 mm) to assess subjective drug effects throughout the study sessions (Farré et al. 2007, Schmid et al. 2015, Holze et al. 2022). We administered 21 VAS items at seven time points during each session: at baseline (0 h), and 0.5 h, 1 h, 1.5 h, 2.75 h, 3.75 h, and 4.25 h post drug/placebo administration. We included four bidirectional VAS items assessing visual perceptual changes along the of dimensions blurrier–sharper, less–more saturated, brighter–darker, more static–more dynamical. The form containing the VAS asked: “How do you currently feel due to the study substance?” Unidirectional VAS items were anchored with “normal” at the lower end of the scale and ‘extreme’ at the upper end of the scale. Bidirectional VAS items featured a vertical line at the center marking “normal” with opposite descriptors (e.g. “I perceive the environment as more static” or “I perceive the environment as more dynamical”) anchored at each end of the scale. Four basic nonspecific drug effect items and the four bidirectional visual effects items are reported in the main text, while the remaining items are provided in the supplement. These items were chosen prior to data analysis to convey a general overview of subjective drug effects. Additionally, the 5D-ASC was administered at the end of every test session to retrospectively rate maximal psychedelic effects.

### Contrast surround suppression task

**Figure 1:**
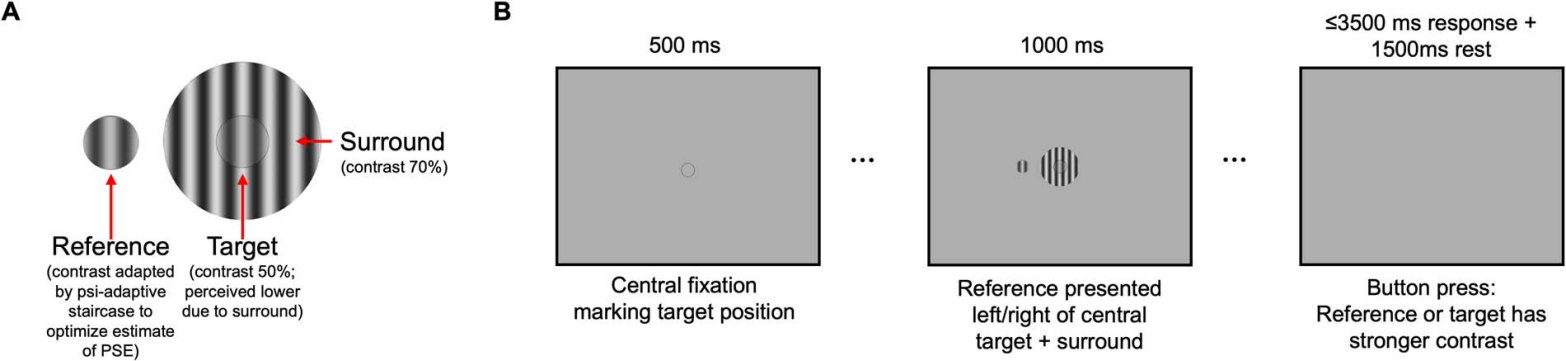
Task paradigm. (A) A reference and target stimulus are concurrently presented. (B) First, a cue marks the location of the target. Subsequently, the stimuli are presented. After, the participant responds by button press and can then rest until the next fixation cue is presented. (PSE = point of subjective equality).

We administered a simple 2AFC task to estimate the reduction of perceived contrast by a high-contrast surround in each session, 4 h after substance administration (Fig. 1). The task was scheduled after a set of EEG-paradigms for logistical reasons. Consequently, the task was not performed during peak plasma levels (~1.5–2 h after ingestion) or peak subjective drug effects (~2.5 h after ingestion). Instead, the task was administered during the plateau phase, where subjective drug effects remain relatively stable while plasma LSD levels have declined to ~60%–80% of the peak (Family et al. 2020, Holze et al. 2021a, 2021b). In the task, two circular drifting sine wave gratings were presented on a grey background for 1000 ms. The gratings drifted at a speed of 1.5 cycles/°, had a spatial frequency of 2 cycles/°, and their orientation angle (0°, 45°, 90°, 135°) and drift direction were randomly drawn for each trial but were the same for both gratings. The “target” grating of 50% Michelson contrast with a radius of 0.66° was presented at the center of the screen. It was surrounded by an annular sine wave grating with 2° outer radius and 70% contrast. A “reference” grating of identical size to the target, but with varying contrast, was presented left or right of the target at 3° center–center eccentricity (see Fig. 1A).

Participants were instructed to fixate on the target, while the reference appeared pseudo-randomly on the left or the right in each trial. A thin dashed circle, marking the outer edge of the target grating, was presented for 500 ms prior to stimulus onset and during stimulus presentation to facilitate fixation. Following stimulus presentation, participants had up to 3500 ms to press the X or N key with their left or right index finger, indicating whether they perceived the left or right grating as having stronger contrast, respectively. After responding, participants could then rest for 1500 ms before the dashed circle indicated the start of the next trial (see Fig. 1B). The task consisted of 100 trials, with a self-paced break after the 50th trial.

The contrast of the reference grating varied from trial to trial according to two interleaved psi-marginal adaptive staircases as implemented in the Palamedes Toolbox (Prins 2013, Prins and Kingdom 2018). These staircases were set to estimate the individual point of subjective equivalence (PSE), defined as the reference contrast perceived equivalent to the target contrast. Since the target contrast was held constant at 0.5, the difference between the PSE and 0.5 served as an index of surround suppression strength. For the staircase configuration, the lapse and guess rate parameters were set to 0.02. The slope parameter was treated as a nuisance parameter and thus “marginalized”, meaning the staircase selected contrast values for upcoming trials exclusively to maximize information gain about the PSE, effectively ignoring uncertainty about the slope.

Participants rested their chins on a chinrest positioned 62 cm from the screen under dim lighting in a psychophysics laboratory. The task was implemented with Psychtoolbox-3 (Kleiner et al. 2007) in MATLAB (The MathWorks Inc 2023) and presented on a screen with linearized luminance. During the screening visit, participants performed a training script introducing the stimuli and concluding after a brief practice block.

### Statistical analyses

For the contrast surround suppression task, we first fitted logistic psychometric functions to each session’s responses using the Palamedes toolbox (Prins and Kingdom 2018), with lapse and guess rate parameters set to 0.02, similar to previous analyses that fixed it at 0.04 (Schallmo et al. 2015, Swanson et al. 2024). From the fits, we derived the PSE, which indicates the subjective contrast of the target. Session data were excluded if the fitted PSE fell outside the range 0–0.5, as such values likely reflected misunderstanding of the task (e.g. comparing the reference grating with the surround, difficulty perceiving the reference, or other performance errors). Participants with more than one excluded session were omitted from group analyses (see Supplementary Methods “Data Exclusion” and Supplementary Fig. 1 for further information on the rationale and statistics on the excluded data). To quantify surround-induced contrast decrement we subtracted the true target contrast (0.5) from the PSE. Then, we used a linear mixed-effects model (LMM) to test the effect of drug condition on contrast decrement using the following model:


\begin{align*} & \mathrm{Contrast}\_\mathrm{dec}\sim \mathrm{DrugCondition}+\mathrm{StudyDay}\\&\quad +\,\mathrm{DrugCondition}:\mathrm{StudyDay}+\left(1\ |\ \mathrm{SubjectID}\right) \end{align*}

Of note, in the preregistration we specified another, more complex model including random slopes. This model could not be fitted to the data because the number of model parameters exceeded the number of available data points. Hence, we do not report any results on that model or the model comparison. The LMM analysis was performed using the lmer function in the R package lme4 (version 1.1-36; Bates et al. 2015). Unspecified in the preregistration, we fitted the model using restricted maximum likelihood estimation and subsequently evaluated statistical significance of the factors using a type-III analysis of variance (ANOVA) with Satterthwaite’s degrees of freedom method, as implemented in the lmerTest package (version 3.1-3; Kuznetsova et al. 2017).

As preregistered, we performed a sensitivity analysis to address potential bias due to missing data. Specifically, we estimated two additional LMMs in which missing data from participants with only two included sessions were imputed using either the maximum or the minimum of the contrast decrement values observed in the sample.

In addition to the registered analyses of the task data, we performed a Bayesian analysis to evaluate whether the data provided evidence in favor of or against the hypothesis that drug condition predicts contrast decrement. In this, we compared the full model to a model excluding drug condition and its interaction with study day. The strength of evidence was quantified using a Bayes factor. The Bayesian analyses were done using the brms package in R (version 2.22.0; Bürkner 2017). Our Bayesian analysis used moderately informative priors, and posterior predictive checks and convergence diagnostics confirmed adequate fit (full specification of priors and more information are given in Supplementary Methods “Bayesian Analysis”).

As preregistered, we tested the association between objectively measured and reported subjective visual effects using a non-parametric Spearman correlation implemented in the rstatix package (version 0.7.2; Kassambara 2019). Specifically, we correlated the difference between the 20 μg and placebo conditions of VAS item 8 “change in visual perception” averaged over the last two VAS timepoints (+3.75 h and + 4.25 h, before and after the task) (∆VAS) with the difference in contrast decrement in the contrast surround suppression task between the same conditions (∆Contrast_dec).

In addition to the preregistered analyses, we performed exploratory analyses to compare select VAS items (“any”, “good”, “bad”, “visual drug effects”, and the specific visual items) and the 5D-ASC dimensions between conditions. VAS items were averaged across measurements obtained between +1.5 and + 4.25 h, capturing the expected window of acute drug effects with available data prior to participant discharge. Averaging across time points increases sensitivity by reducing measurement noise, including that arising from expectancy or procedural factors. This approach is particularly relevant in low-dose studies where maximal values are more susceptible to placebo-related confounds. For the 5D-ASC, we evaluated each dimension’s percent of the dimension maximum. Because both the VAS and the 5D-ASC data are bounded and contain a high count of zero values, we used ordered beta regression to test whether there was an effect of drug condition on subjective effect reports. Ordered beta regression is designed specifically for semi-continuous data that are bounded at both ends, but are continuous in between, such as percentage, VAS, or slider data. We implemented the analyses using the ordbetareg package in R (version 0.8; Kubinec 2023), which fits a mixed Bayesian regression under the ordered beta distribution. The model formulation mirrored the structure used for the task data analyses:


\begin{align*} & \mathrm{VAS}/5\mathrm{D}-\mathrm{ASC}\ \mathrm{dimension}\sim \mathrm{DrugCondition}+\mathrm{StudyDay}\\&\quad +\,\mathrm{DrugCondition}:\mathrm{StudyDay}+\left(1\ |\ \mathrm{SubjectID}\right) \end{align*}

Subsequently, we estimated average marginal effects using the marginaleffects package (version 0.27.0; Arel-Bundock et al. 2024). Here, average marginal effects represent the expected change in the outcome for a specified contrast between conditions, averaged across all participants (with subject-level random intercepts integrated out) and over the distribution of covariates (Arel-Bundock et al. 2024, Rohrer and Arel-Bundock 2025). This approach is particularly helpful for interpreting the results of nonlinear models, like ordered beta regression, where differences in the coefficients on the link scale do not translate straightforwardly onto the outcome scale (Kubinec 2023, Iannario et al. 2024, Scholbeck et al. 2024, Rohrer and Arel-Bundock 2025). We considered effects credible if the 95% credible interval of the effect estimate did not include zero.

For the bidirectional VAS, we first performed a binomial test to evaluate whether non-zero values were symmetrically distributed between the positive and the negative ends of the scale across conditions. When a statistically significant difference was found, we set the values of the less frequently reported direction for that item to zero and modeled the remaining data with ordered beta regression. This approach assumed that any effect would have a consistent direction and then tested for condition effects in the identified direction with the ordered beta regression.

## Results

### Sample

We screened 41 healthy volunteers to include a total of 33 participants, of whom three were replacements for premature dropouts unrelated to the study procedures. Of the dropouts, one completed two appointments, one completed one appointment, and one completed only the screening visit, resulting in 30 participants (15 female) who completed the study. Consistent with our preregistration, analyses included all participants who completed at least two sessions, yielding a final sample of 31 participants (15 female).

Most participants reported regular consumption of alcohol (96.8%) and caffeine (87.1%) as well as lifetime cannabis use (83.9%). About half of the participants also reported lifetime consumption of MDMA, sedatives, stimulants, and psychedelics. Detailed sample characteristics of the 31 participants who were included in the analyses are presented in Table 1.

**Table 1:** Sample characteristics.

Demographics			
		Mean (SD)	Range
Age		28.1 (8.12)	18–52
BMI		22.5 (2.08)	18–26
Sex	48% female		
**Legal Drug Use**			
**Drug**	**Current**	**Units/week**	**Units/week**
	**consumption n (%)**	**Mean (SD)**	**Range**
Alcohol	30 (96.8%)	2.1 (2)	0.23–9
Caffeine	27 (87.1%)	13.2 (7.5)	1–35
Nicotine	5 (16.1%)	22.4 (14.3)	7–35
**Illegal Drug Use**			
**Drug**	**Lifetime consumption**	**Lifetime consumption occasions**	**Lifetime consumption occasions**
	**n (%)**	**Mean (SD)**	**Range**
Cannabis	26 (83.9%)	264 (857)	1–4 500
MDMA	15 (48.4%)	9.2 (13.6)	1–50
Sedatives	16 (51.6%)	6.5 (7.8)	1–30
Stimulants	15 (48.4%)	11.9 (22.6)	1–80
Opiates	1 (3.2%)	1 (−)	1
Psychedelics	16 (51.6%)	5.3 (4.5)	1–17
Other drugs	1 (3.2%)	5 (−)	5

Characteristics and drug consumption of the participants that completed two or more sessions (*n* = 31). SD = standard deviation, BMI = body mass index, MDMA = Methylenedioxymethamphetamine.

### Contrast surround suppression

**Figure 2:**
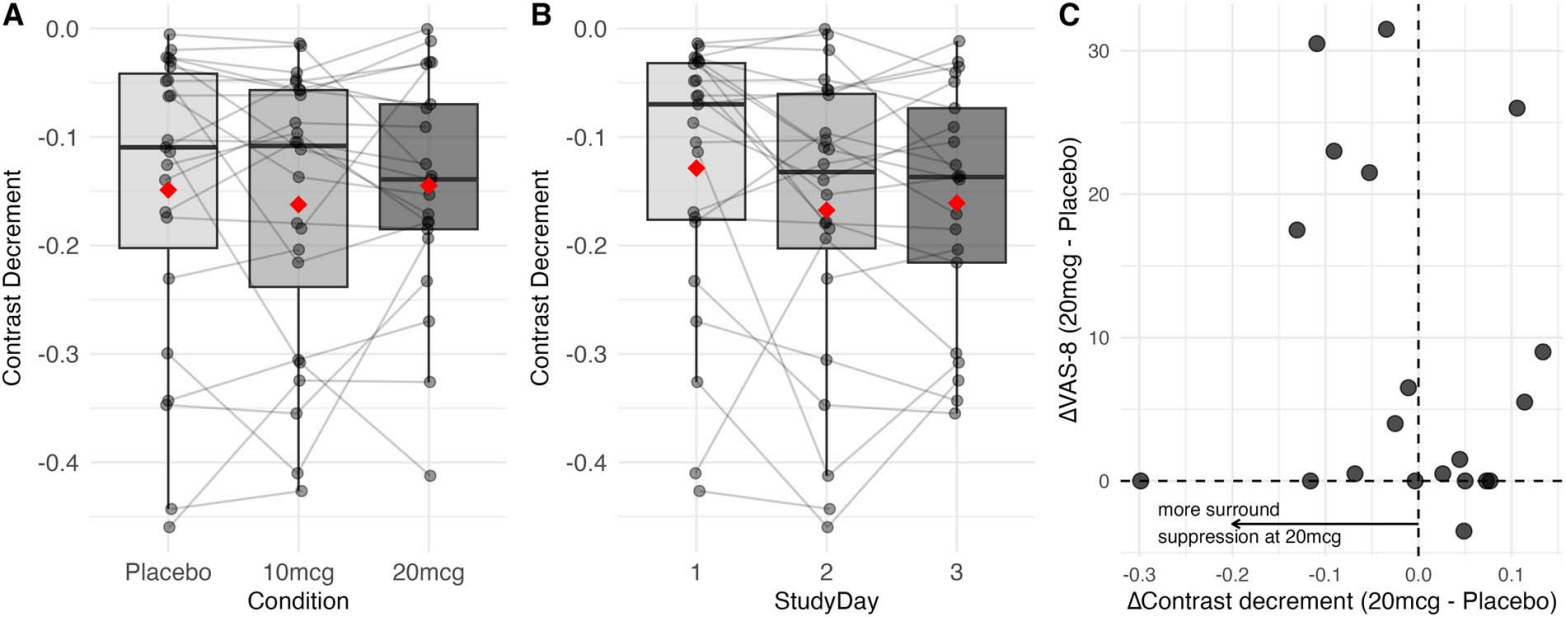
Contrast surround suppression. (A) Surround-induced contrast decrement per drug condition. (B) Contrast decrement per session. Boxplots display the median (horizontal line), interquartile range (IQR; box boundaries), and minimum/maximum values within 1.5 × IQR (whiskers). Dots represent individual participant values, with grey lines connecting repeated measures across conditions/sessions. Red diamonds indicate the mean of each condition/session. (C) Non-significant correlation of ∆VAS-8 and ∆contrast decrement.

Following the session exclusion criteria, we report data from 68 of 93 completed sessions across 24 participants. Missing session rates in the included data did not differ significantly between drug conditions (see Supplementary Methods “Data Exclusion” for further information and statistics on the data exclusion). To evaluate H1, we fit an LMM with fixed effects for DrugCondition, StudyDay, and their interaction, and a random intercept for SubjectID. Boxplots of contrast decrement per drug condition and study day are shown in Fig. 2A and B. The model intercept was negative and statistically significant (*b* = −0.15, *SE* = 0.02, *t*(22.45) = −6.38, *P* < .001), indicating the task elicited a significant contrast surround suppression effect on average. However, the type-III ANOVA found no significant main effect of drug condition (*F*(2, 36.87) = 0.47, *P* = .628), study day (*F*(2, 36.76) = 1.47, *P* = .242) or their interaction (*F*(4, 43.22) = 1.23, *P* = .313; see Table 2; fixed and random effects estimates in Supplementary Table 1). We reproduced this null result in the subgroup of psychedelic-naïve participants (*n* = 10 participants with included data from at least two sessions; *F*(2, 15.06) = 1.16, *P* = .34; Supplementary Tables 2–3).

**Table 2:** Type-III ANOVA contrast surround suppression.

	SS	MS	Numerator df	Denominator df	F	*P*
DrugCondition	0.003	0.002	2	36.87	0.47	.628
StudyDay	0.010	0.005	2	36.76	1.47	.242
DrugCondition: StudyDay	0.017	0.004	4	43.22	1.23	.313

SS = Sum of Squares, MS = Mean Squares, df = Degrees of Freedom. Denominator df were estimated using Satterthwaite’s method.

The sensitivity analysis using minimum and maximum imputation of missing data corroborated the null results of the main analysis (Supplementary Tables 4–7; Supplementary Fig. 2). Given the null result, we also conducted a Bayesian analysis comparing the full model to a reduced model without drug condition and its interaction with study day as predictors. The Bayes factor of 0.005 indicated very strong evidence against an effect of drug condition on contrast surround suppression.

Analysis of average reaction times in the included data revealed a trend effect of condition (*F*(2, 37.3) = 2.87, *p* = .069; Supplementary Tables 8–9). Uncorrected pairwise t-tests point to an increase in reaction times after 20 μg (mean = 436 ms) relative to placebo (mean = 359 ms; *P* = .037, other pairwise *P*s > .05), which did not survive correction for multiple comparisons. Separate analyses of the responses to stimuli from the two interleaved staircases suggest overall high reliability as the correlation between the PSE from either set of trials was high across conditions (Placebo: ρ = 0.93; 10 μg: ρ = 0.89; 20 μg: ρ = 0.85; all *Ps* < .001).

Finally, to test H2, we assessed the correlation between difference in contrast decrement between placebo and 20 μg (∆Contrast_dec) and the corresponding difference in subjective visual effects (∆VAS-8) using Spearman correlation. No significant association was found (ρ(18) = −0.15, *P* = .519; Fig. 2C).

### Subjective drug effects

**Figure 3:**
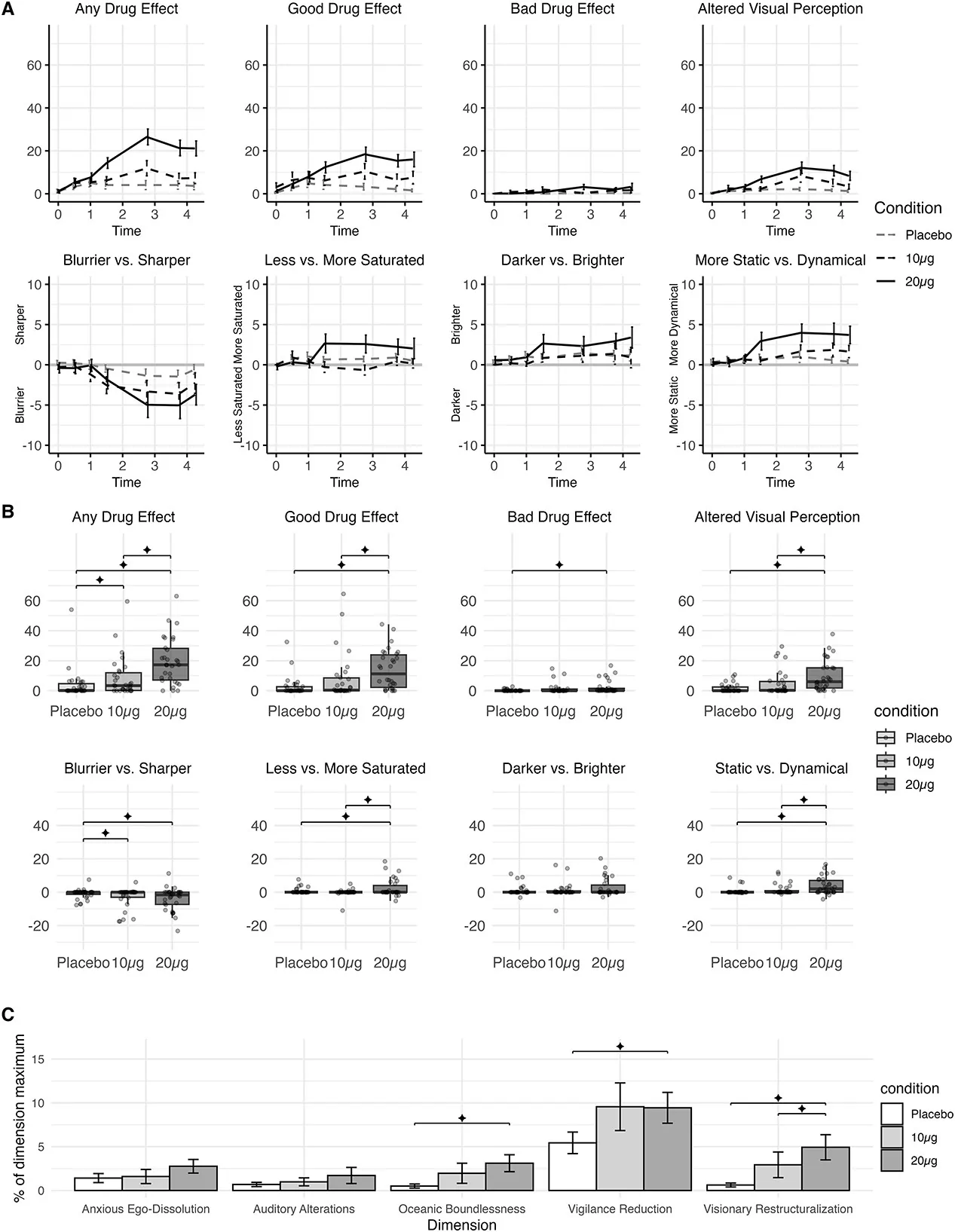
Subjective effects. (A) Mean time-courses of visual analog scales (VAS) assessing subjective drug effects. Error bars indicate the standard error of the mean. (B) Mean of VASs assessing subjective drug effects over measurements from +1.5 h − +4.25 h. (C) Percent of the dimension maximum of each 5D-ASC dimension per condition. VAS = visual analog scale, 5D-ASC = 5-dimensional altered state of consciousness rating scale. ✦ indicates a credible condition difference (95% credible interval of the average marginal effect excludes zero; see methods for model details and Table 3 for statistics for all contrasts).

Figure 3 shows the time courses (Fig. 3A) and average ratings between +1.5 h and +4.25 h (Fig. 3B) of selected nonspecific drug effects and specific visual effects. Table 3 presents the average marginal effect estimates from the ordered beta regression with 95% credible intervals (CI). Effect estimates describe the shift of the mean of the ordered beta distribution on a scale from 0–100 mm for unidirectional VAS items and from 0 to ±50 mm for bidirectional VAS items. The 95% CIs are given alongside the marginal effect estimates in square brackets in the main results below. Marginal effects for the remaining VAS items obtained in the study are reported in Supplementary Table 10.

**Table 3:** Subjective effects average marginal effect of drug condition.

VAS Item/5D-ASC Dimension	10 μg—Placebo		20 μg—Placebo		20 μg—10 μg	
	Estimate	95% CI	Estimate	95% CI	Estimate	95% CI
Selected VAS Items						
VAS-1: “Any drug effect”	**4.933**	**[0.880, 9.412]**	**17.124**	**[11.418, 23.185]**	**12.150**	**[5.919, 18.413]**
VAS-2: “Good drug effect”	4.268	[−0.056, 8.785]	**11.946**	**[6.867, 17.553]**	**7.697**	**[2.052, 13.677]**
VAS-3: “Bad drug effect”	0.746	[−0.656, 2.386]	**2.262**	**[0.643, 4.979]**	1.523	[−0.218, 3.972]
VAS-8 “Altered visual perception”	2.574	[−0.112, 5.552]	**7.510**	**[4.233, 11.536]**	**4.956**	**[1.272, 8.984]**
VAS-18: “Blurrier *vs.* sharper”	**−3.665**	**[−6.914, −0.877]**	**−7.222**	**[−11.338, −3.724]**	−3.555	[−7.705, 0.716]
VAS-19: “Less *vs.* more saturated”	−0.919	[−3.100, 0.364]	**2.628**	**[0.381, 6.132]**	**3.602**	**[1.384, 7.589]**
VAS-20: “Darker vs. brighter”	0.590	[−1.442, 3.382]	2.212	[−0.005, 5.336]	1.569	[−1.331, 4.847]
VAS-21: “Static vs. dynamical”	1.250	[−0.180, 3.358]	**3.655**	**[1.862, 6.970]**	**2.388**	**[0.454, 5.296]**
5D-ASC Dimensions						
Oceanic Boundlessness	1.159	[−0.731, 3.723]	**2.218**	**[0.252, 5.134]**	1.044	[−1.592, 3.949]
Anxious Ego-Dissolution	−0.315	[−2.192, 1.624]	1.317	[−0.441, 3.489]	1.634	[−0.408, 4.055]
Visionary Restructuralization	1.440	[−0.713, 4.033]	**4.745**	**[2.024, 8.690]**	**3.294**	**[0.190, 7.209]**
Auditory Alterations	0.272	[−1.172, 2.033]	0.811	[−0.667, 2.750]	0.541	[−1.276, 2.503]
Vigilance Reduction	3.643	[−0.061, 7.401]	**4.601**	**[0.893, 8.547]**	0.942	[−3.233, 5.351]

Average marginal effects represent the expected change in the outcome for the specified contrast, averaged across participants (with random intercepts integrated out) and over the distribution of covariates. For instance, the upper left value gives the average increase in ratings of any drug effect after administration of 10 μg compared to placebo*.* Estimates and 95% credible intervals (CI) are in bold where the CI does not include zero and hence, we interpret the estimated marginal effect to be credibly different from 0 on average. VAS = Visual Analog Scale; 5D-ASC = 5-Dimensional Altered State of Consciousness Rating Scale.

Participants rated all unidirectional VAS items higher after 20 μg compared to placebo. Specifically, ratings increased from placebo to 20 μg as follows: “any drug effect” by 17.12 [11.42, 23.19], “good drug effect” by 11.95 [6.87, 17.55], “bad drug effect” by 2.26 [0.64, 4.98], and “altered visual perception” by 7.51 [4.23, 11.54]. Ratings of “any drug effect” were also higher after 10 μg compared to placebo by 4.93 [0.88, 9.41]. Furthermore, dose escalation from 10 μg to 20 μg increased ratings of “any drug effect” by 12.15 [5.92, 18.41], of “good drug effect” by 7.69 [2.05, 13.68], and of “altered visual perception” by 4.96 [1.27, 8.98].

For the bidirectional VAS item measuring blurrier vs. sharper perception, participants reported increased blurriness, with 10 μg enhancing blurriness by −3.67 [−6.91, −0.88] and 20 μg by −7.22 [−11.34, −3.72] on average compared to placebo. This pattern of larger point estimates in the drug dose ratings contrasted with placebo suggests a trend toward monotonically blurrier perception, although the 20–10 μg contrast was not credible at −3.55 [−7.71, 0.72]. Additionally, participants reported their visual fields were more saturated by 2.63 [0.38, 6.13] and more dynamical by 3.65 [1.86, 6.97] on average after 20 μg compared to placebo. Moreover, increased dose from 10 μg to 20 μg enhanced ratings of saturation by 3.60 [1.38, 7.59] and ratings of dynamicity by 2.39 [0.45, 5.30].

Of the 5D-ASC dimensions, Oceanic Boundlessness (OB) increased by 2.22% [0.25, 5.13], Visionary Restructuralization (VisR) increased by 4.75% [2.02, 8.69], and Vigilance Reduction (VigR) increased by 4.60% [0.89, 8.55] of the dimension maximum on average in the 20 μg condition compared to placebo. Additionally, VisR items were rated higher after 20 μg compared to 10 μg by 3.29% [0.19, 7.21]. No credible differences were found for the remaining 5D-ASC dimensions.

We also computed average marginal effects for the 11-factor rescoring of the 5D-ASC (see Supplementary Table 11), providing more nuanced insight into the affected dimensions. For instance, of the factors related to visual alterations, Complex Imagery was not rated credibly higher after 20 μg compared to placebo (0.75% [−0.93, 3.51]), but Elementary Imagery (3.39% [0.51, 8.51]), Audio-Visual Synesthesia (6.33% [2.77, 10.36]), and Changed Meaning of Percepts (2.89% [0.42, 6.67]) were. Moreover, the factor Impaired Control and Cognition (2.71% [0.90, 5.88]) was enhanced after 20 μg compared to placebo, but not the factor Anxiety (1.87 [−0.01 4.13]). We did not find changes in ratings of Spiritual Experience (0.86% [−1.29, 3.99]) or Experience of Unity (1.37% [−1.32, 4.17]), but heightened Blissful State (3.51% [0.59, 8.12]) and Insightfulness (3.98% [1.12, 8.31]) after 20 μg compared to placebo. Lastly, we found dose-dependently higher experience of disembodiment from placebo to 10 μg (1.91% [0.31, 4.10]), Placebo to 20 μg (8.58% [5.17, 13.34]), and 10 μg to 20 μg (6.65% [3.05, 11.16]).

Additionally, Supplementary Tables 12–14 present the average marginal effects of study day and average conditional effects of treatment effect difference by study day on the selected VAS items and on the 5D-ASC. Notably, the VAS items “Altered visual perception” (−5.06 [−8.77, −1.69]) and “Blurrier vs. sharper” (4.51 [0.89, 8.59]) were rated less extremely on the last compared to the first study day. Similarly, the three 5D-ASC dimensions affected by LSD were rated lower on the last compared to the first study day (OB: −2.46 [−5.76, −0.36]; VisR: −2.79 [−6.41, −0.07]; VigR: −5.29 [−9.51, −1.01]; see Supplementary Table 12).

The conditional average treatment effects by study day revealed differences in the ratings of multiple VAS items and 5D-ASC dimensions within the 10 μg condition. Specifically, multiple items and dimensions were rated lower on the second and third compared to the first study day with some also credible reductions on the third relative to the second day in the 10 μg condition. Additionally, we found credibly brighter perception on the third relative to the second day 2.79 [0.38, 8.20] and less dynamical perception on the second relative to the first day −1.49 [−4.99, −0.19] in the placebo condition (see Supplementary Tables 13 and 14 for detailed statistics).

### Blinding integrity

Blinding was assessed at the end of each session by asking participants to indicate which treatment they believed they had received during that session. At the end of the study, participants were again asked to specify during which sessions they believed they had received which treatment. Overall, participants correctly guessed their treatment in 54% of sessions and in 64% of cases at the end of the study, which is both significantly above the 33.3% chance level (binomial test; both *P* < .001). This was primarily due to correct identification of the placebo vs. 20 μg sessions. Placebo was correctly identified by 63% at session end (*P* < .001) and by 70.0% at study end (*P* < .001), and the 20 μg by 61% at session end (*P* = .002) and by 73% at study end (*P* < .001). The 10 μg-condition, in contrast, was not correctly identified above chance level at either time point (session end: 38%, *P* = .569; study end: 50%, *P* = .079).

At the end of the sessions, 73% of participants guessed one of the LSD conditions when they had received LSD which was not above the chance level 66.7% (*P* = .349). Conversely, at the end of the study, 85% guessed one of the LSD conditions after receiving active treatment, which was significantly above chance level (*P* = .002). When receiving the 20 μg dose, participants guessed one of the LSD conditions above chance level by 87% at the session end (*P* = .013) and by 93% at study end (*P* = .001), whereas the 10 μg dose was not identified as LSD above chance level at either time point (both *P*s > .05). Complete data about guesses and guess correctness per condition and timepoint and binomial test results are given in Table 4.

**Table 4:** Treatment guess accuracy at end-of-session and end-of-study.

Condition	Sessions	Correctly guessed treatment		Correctly guessed active treatment		Guessed Placebo	Guessed 10 μg	Guessed 20 μg
	n	n (%)	*P vs.* chance	n (%)	*P vs.* chance	n (%)	n (%)	n (%)
Guess at end of the session								
Placebo	30	**19 (63.3%)**	**< .001**			19 (63.3%)	10 (33.3%)	1 (3.3%)
10 μg	31	12 (38.7%)	.569	18 (58.1%)	.342	13 (41.9%)	12 (38.7%)	6 (19.4%)
20 μg	31	**19 (61.3%)**	**.002**	**27 (87.1%)**	**.013**	4 (12.9%)	8 (25.8%)	19 (61.3%)
Guess at end of study								
Placebo	30	**21 (70%)**	**< .001**			21 (70%)	9 (30%)	0 (0%)
10 μg	30	15 (50%)	.079	23 (76.7%)	.333	7 (23.3%)	15 (50%)	8 (26.7%)
20 μg	30	**22 (73.3%)**	**< .001**	**28 (93.3%)**	**.001**	2 (6.7%)	6 (20%)	22 (73.3%)

n(%) = number (percent) of guesses. Bold values: significant binomial test (two-sided, α = 0.05) against chance levels. Chance levels: 33.3% exact condition, 66.6% active treatment. Correct active treatment guesses correspond to guessing one of the two LSD conditions when participants did indeed receive LSD.

## Discussion

This study investigated the effect of low doses of LSD on visual contrast surround suppression in healthy volunteers, using a randomized, double-blind, within-subject crossover design. Our results provide no evidence that LSD alters surround suppression at the doses tested. The Bayesian analysis further supported this absence of effect, providing strong evidence against drug condition predicting changes in surround suppression. However, exploratory analyses revealed changes in subjective visual perception after low-dose LSD. Specifically, participants reported that their visual experience was blurrier, more saturated, and more dynamical, particularly after the 20 μg dose, with blurriness also noted after 10 μg. Additionally, the 20 μg dose increased ratings on several nonspecific VAS items and the 5D-ASC dimensions Visionary Restructuralization, OB, and Vigilance Reduction. Together, these findings suggest that while low-dose LSD does not produce objectively measurable changes in visual contrast surround suppression, it significantly alters subjective visual perception and induces a mildly altered state of consciousness.

### No effect of low-dose lysergic acid diethylamide on contrast surround suppression

The absence of detectable effects of LSD on surround suppression contrasts with previous findings that intermediate and high-dose psilocybin enhance context-dependent visual biases. A pilot study found that 25 mg oral psilocybin enhanced contrast surround suppression in a task akin to ours (Swanson et al. 2024). Another study found that 5 and 10 mg of psilocybin enhanced the Ebbinghaus illusion, a size perception bias driven by visual context (Aqil et al. 2025). Despite our study’s larger sample size compared to the two previous studies, no such effect emerged here, likely reflecting differences in dose, compound, testing time relative to peak effects, and task parameters.

The low doses used are a likely explanation for the absence of an effect of LSD on surround suppression in our study. According to a direct comparison study, LSD base and psilocybin are approximately dose-equivalent at ~1:200 (Holze et al. 2022). On this basis, our higher dose of 20 μg of LSD would correspond to 4 mg of psilocybin and is thus below the 5 mg psilocybin dose reported by Aqil et al. (2025) to enhance the Ebbinghaus illusion. This dose gap may account for the null effect on spatial contextual computations. Notably, participants still reported low-dose LSD to alter subjective visual qualities, suggesting that doses insufficient to shift contrast surround suppression may nonetheless modulate subjective visual perception. Future studies should examine whether higher doses of LSD affect contrast surround suppression or related spatial contextual effects on perception.

Qualitative differences between the pharmacological profiles of psilocybin and LSD may also contribute to the absence of an effect. Both drugs primarily act via agonism at the 5-HT2A receptor (Carter et al. 2007, Kometer et al. 2013, Rickli et al. 2016, Preller et al. 2017, Vollenweider and Preller 2020, Holze et al. 2021b, Becker et al. 2022). At dose-equivalent levels they yield broadly comparable subjective visual effects in humans (Holze et al. 2022, Ley et al. 2023). However, LSD also exhibits partial agonism at dopaminergic D2 receptors, whereas psilocin, the active metabolite of psilocybin, additionally inhibits the serotonin transporter, albeit with low affinity compared to 5-HT2A receptor (Halberstadt and Geyer 2011, Rickli et al. 2016). In rodents, the dopaminergic effects of LSD emerge later than its serotonergic effects (Marona-Lewicka et al. 2005, Marona-Lewicka and Nichols 2007). Although biphasic effects of LSD have not been demonstrated in humans, low-dose LSD has altered behavior in a temporal interval reproduction task administered late in the time course of LSD effects in a manner consistent with dopaminergic engagement, and many studies point to stimulant-like effects of low-dose LSD (Yanakieva et al. 2019, Hutten et al. 2020, 2024, de Wit et al. 2022). Notably, in schizophrenia, a condition associated with elevated D2-dependent neurotransmission, contrast surround suppression is reduced or even reversed (Yoon et al. 2010, Tibber et al. 2013, Schallmo et al. 2015). It is thus conceivable that later dopaminergic actions of LSD, occurring during our task administration window, could counteract any 5-HT2AR-mediated enhancement of surround suppression. This interpretation remains speculative but highlights testable hypotheses. Future studies examining the effects of LSD on surround suppression could manipulate assessment time points and/or pre-treat participants with receptor-selective antagonists to dissociate serotonergic from dopaminergic contributions to contextual visual computations.

Study procedures may also have contributed to the null effect. The task was administered four hours post-dose after multiple other tasks, potentially introducing fatigue. This may have been exacerbated by caffeine withdrawal. While we required participants to refrain from caffeine consumption to control for its potential confounding of LSD’s effects on neurophysiological measures and surround suppression (Nguyen et al. 2018), withdrawal in regular consumers may have inadvertently negatively impacted attention and executive function, and increased fatigue during the task. Elevated scores on the Vigilance Reduction dimension of the 5D-ASC across drug conditions are consistent with reduced alertness. Average reaction times showed a trend toward an effect of condition; pairwise comparisons indicated slower responding after 20 μg LSD compared to placebo. While this may suggest reduced task engagement after LSD, the effect did not remain statistically significant after correcting for multiple comparisons. Lastly, the task was not administered during peak drug effects. Future studies should assess contrast surround suppression closer to peak LSD effects and minimize pre-task load.

Task design choices may have also contributed to the null effect. We used a 2AFC task variant with central target and lateral reference, rather than bilateral presentation of reference and target on opposite sides of a central fixation (Schallmo et al. 2015, Swanson et al. 2024). This variant yielded more reliable results in piloting without drug. One could speculate that LSD differentially affects peripheral and central contrast perception, potentially obscuring effects of LSD on contrast surround suppression in our design. However, prior work suggests that psychedelics impact top-down perceptual processes rather than early sensory processing such as basic contrast perception (Carter et al. 2004). It thus seems unlikely that any unknown effects of LSD on peripheral vs. central contrast perception masked an effect of LSD on surround suppression in our paradigm.

We observed strong contrast surround suppression (15% on average) among included participants. Internal reliability, as measured by the correlation of PSE values estimated from trials from the two interleaved staircases, was high across conditions in included participants. Excluded participants typically showed random or heavily biased choice patterns, consistent with misinterpretation of the instruction or inability to perform the task (see Supplementary Fig. 1). Thus, the paradigm appears suitable for eliciting contrast surround suppression and for detecting drug-induced changes.

Only ~ 74% of study sessions met the registered inclusion criteria, indicating that a subset of participants struggled with the task, potentially due to fatigue or task demands. Similar exclusion criteria are common in psychophysical studies of contextual and illusion phenomena (Schallmo et al. 2015, Dogge et al. 2018, Leptourgos et al. 2020). Although “adequate task performance” was an inclusion criterion for the study, this was based solely on performance in the other tasks, which entailed clear, objective cut-off measures, such as choice accuracy in training blocks. Future work could prospectively apply a quality metric like the PSE range criterion, as applied here after data acquisition, to reduce post hoc data loss.

### Low-dose lysergic acid diethylamide induces mild subjective visual perceptual and psychedelic-like effects

In contrast to the null results in the contrast surround suppression task, we observed clear and consistent nonspecific subjective drug effects as well as specific effects on subjective visual perception. As for the nonspecific effects, LSD dose-dependently enhanced ratings of “any drug effect” and 20 μg of LSD enhanced ratings of “good drug effect”, “bad drug effect”, and “visual drug effect” compared to placebo. In addition to the effect on the VASs, we found an effect of condition on three of the five dimensions of the 5D-ASC. Specifically, we observed higher scores on the dimensions Visionary Restructuralization, OB, and Vigilance Reduction for 20 μg compared to placebo. These results jointly suggest that 20 μg of LSD base induces a mildly altered state of consciousness.

Our results on general subjective drug effects corroborate previous research into low-dose LSD that found similar effects (Bershad et al. 2019, Hutten et al. 2020, Holze et al. 2021a, 2021b, de Wit et al. 2022, Murray et al. 2022). There are some notable differences to these previous studies, however. For instance, we observed numerically higher average ratings of “any drug effect” for the 20 μg dose than were found for a 25 μg dose in a previous study using identical VASs (Holze et al. 2021b). This is likely because of a scale anchoring effect due to the comparison conditions, as in that study, the comparator conditions were high-dose LSD and placebo, whereas our study compared 20 μg to an even lower dose and placebo. Also, there is some heterogeneity in which 5D-ASC dimensions are affected or which factors of the alternative 11-ASC scoring method were altered. For example, in a previous study, a dose of 26 μg LSD tartrate only significantly affected blissful state, a subfactor of the OB dimension, while the other dimensions showed qualitative dose-ordered increases but were not significantly affected by dose in this study (Murray et al. 2022). Another study found significant effects of 20 μg LSD hydrate on the same dimensions that were affected in our study and on the dimension Anxious Ego-Dissolution (Hutten et al. 2020). Of note, in our study, the ratings of Reduction of Vigilance across drug conditions and the credible effect of 20 μg compared to placebo may be modulated by the psychophysics laboratory environment and the overall study procedures, which may have increased fatigue. Other differences between studies may be due to subtle differences in doses and formulations, lower sample sizes in previous studies, and the use of statistical analysis methods not tailored to bounded data.

Due to our interest in visual perception, we also obtained reports on multiple bidirectional VAS items inquiring about specific alterations in visual perception. These showed that after 20 μg, participants perceived their surroundings as blurrier, more saturated, and more dynamical compared to placebo. Notably, participants also reported blurrier vision after 10 μg. And, although the marginal effect estimate comparing blurriness at 20 μg to 10 μg was negative and the 95% credible interval was largely negative, it was not reliably different from zero. This pattern suggests a trend toward monotonically increased blurriness under LSD, even if the difference between the two active doses was not statistically conclusive. These results from the specific visual VAS items complement the enhanced ratings of the Visionary Restructuralization dimension and the related 11ASC factors, as well as the VAS item “altered visual perception”. Taken together, they suggest that basic visual perceptual alterations occur after LSD, even at mildly psychoactive doses that do not cause a classic psychedelic experience.

In addition to the effects of drug condition, we evaluated the effects of order and of the drug condition on different study days on the subjective drug effects. We found some indication that subjective effects were rated less extremely on the last day when averaging over drug conditions, consistent with familiarization or anchoring. Decreases as a function of session appeared most prominent in the 10 μg condition on later study days compared to the first day, potentially reflecting context-dependent scaling based on having experienced other drug conditions, likely especially the higher 20 μg dose.

The observed drug effects could in part be driven or enhanced by expectancy/placebo. In particular, reports of subjective visual alterations could be due to activated expectancies in the context of a study investigating visual perception. A previous study found that the effects of psilocybin microdosing on awe and esthetic experiences were explained by expectancy effects (van Elk et al. 2022). Regarding the bidirectional scales we used to assess subjective visual effects, a placebo effect in the form of an activated expectancy bias, as in the psilocybin study, would require a specific expected direction. We doubt that all participants held clear expectations about the direction in which LSD administration would change their perception on the bidirectional VAS items. In the case of the VAS item “blurrier vs. sharper,” one may even hypothesize an expectation opposite to the observed increased blurriness, as some informational sources cite enhanced visual acuity and sharper perception as expected effects of low-dose psychedelics (e.g. https://effectindex.com). Lastly, in the presence of strong activated expectancy, experiencing some visual alteration should lead to strong effects across visual effect scales. For the 10-μg dose we found no above-chance unblinding but a single credible visual effect on blurriness, while for the higher dose there was considerable heterogeneity in how strongly the different visual VAS items were affected. Coupled with the directionally specific, dose-ordered blurriness effects vs. placebo, these observations suggest that expectancy alone is unlikely to fully explain the subjective visual alterations.

Nevertheless, examining expectancy and blinding integrity is pertinent. Pre-trial expectancy was not measured, but multiple participants mentioned expecting visual effects during the structured screening interview. Treatment-guess data indicates that participants could identify 20 μg and placebo above, but 10 μg only at chance level, both at the end of the session and at the end of the study. Moreover, at session end, participants did not guess one of the LSD doses above chance when having received LSD. Because placebo and 20 μg were identified above chance level, expectancy may have inflated subjective effect ratings, nonetheless. Although not used to address expectancy, the use of bidirectional VAS items makes a purely expectancy-based account less likely. Moreover, blurriness and some showed dose ordering of the effects, where 10 and 20 μg were larger than placebo, and the point estimate of 20 was about double that of 10 μg (though the 20–10 μg contrast was not credibly different from zero), which is hard to explain by expectancy alone. Taken together, we interpret the findings to suggest that at least part of the subjective visual alterations reflect genuine drug-related effects.

### Subjective drug effects in absence of objectively measured task-based effects

Various explanations may account for the discrepancy between robust subjective visual alterations and the absence of detectable effects of low-dose LSD on contrast surround suppression. First, methodological and design factors may have limited sensitivity to drug effects on the task. Nevertheless, the task robustly induced surround suppression. Second, session exclusions reduced power for the task analyses relative to the subjective analyses, potentially lowering sensitivity to small effects; even so, our results, taken together, and especially the Bayesian analysis were consistent with a null effect at the tested doses. Third, one may object that the subjective effects could be inflated by expectancy/placebo. However, the subjective effects broadly align with previous research on low-dose LSD and persisted under partially effective blinding, suggesting that they are unlikely to be fully explained by expectancy alone. Accordingly, the dissociation of altered visual experience without concomitant surround suppression may inform our understanding of visual processing under psychedelics.

Surround suppression is commonly modeled as comprising an early, orientation-insensitive component and a later orientation-sensitive mechanism. The early component is linked to LGN–V1 suppression and the latter to recurrent excitation in V1, lateral V1 connections, and feedback from higher visual areas (Webb et al. 2005, Angelucci and Bressloff 2006, Nassi et al. 2013, Nurminen and Angelucci 2014, Schallmo and Murray 2016, Schallmo et al. 2019). Human evidence remains mixed regarding which component psychedelics may influence. While, contrast surround suppression after psilocybin was not modulated by orientation, EEG data from five participants suggests involvement of the later, orientation-sensitive cortical mechanism in mediating contrast surround suppression (Schallmo et al. 2019, 2025). Additionally, human visual cortex fMRI responses to drifting checkerboards after psilocybin show a pattern of surround suppression more consistent with activation of 5-HT1A, rather than 5-HT2A receptors, according to predictions from chemoarchitectural population receptive field modeling (Aqil et al. 2024, 2025). By comparison, DOI, which is markedly more 5-HT2A receptor-selective than LSD and psilocybin, reduces surround suppression in mice (Ray 2010, Halberstadt and Geyer 2011, Michaiel et al. 2019), underscoring a potentially complex interplay of 5-HT1A and 5-HT2A receptors in modulating surround suppression. Taken together, these observations from distinct assays and species implicate recurrent and cortico-cortical pathways in psychedelic modulations of surround suppression while leaving the receptor-level drivers uncertain.

Leading accounts of psychedelic phenomenology emphasize altered hierarchical inference, either by reducing the precision of priors at higher cortical areas and thereby weakening top-down inhibition (Carhart-Harris and Friston 2019), or by reducing thalamic gating and thereby increasing sensory throughput (Vollenweider and Geyer 2001). Moreover, at low doses, LSD has repeatedly been shown to induce stimulant-like effects at least as reliably as psychedelic-like effects (Hutten et al. 2020, 2024, de Wit et al. 2022, Murray et al. 2022), indicating putative dopaminergic contributions that could additionally shift arousal and precision-weighting without perturbing early visual contextual gain control. These mechanisms together provide plausible routes to altered subjective experience without changes in contrast surround suppression.

Thus, a parsimonious account of the dissociation of experience and behavior is that low-dose LSD modulates distributed feedback, arousal, and precision-weighting processes to cumulatively produce subjective effects, while not measurably altering early contextual gain indexed by surround suppression. To test this, future studies should vary assessment time points relative to the putative distinct pharmacological phases and evaluate paradigms that index more distributed control processes, such as evidence accumulation and decision formation, where serotonergic and potentially dopaminergic modulation could exert cumulative effects.

## Conclusion

In sum, the preregistered analyses showed no detectable effect of low-dose LSD on contrast surround suppression, whereas exploratory analyses indicated credible effects on subjective visual perception. The absence of a task effect is consistent with a null effect of LSD on surround suppression at the tested doses and assessment time point. Higher doses or earlier assessment might reveal effects, and pharmacological differences from psilocybin cannot be excluded. Subjective reports indicate that low-dose LSD induces a mildly altered state of consciousness and alters visual perception in specific dimensions. Specifically, participants reported higher scores on several 5D-ASC dimensions, as well as more saturated and more dynamical perception at 20 μg, and LSD increased blurriness of perception from placebo to 10 and 20 μg. This dissociation of altered subjective experience without concomitant changes in surround suppression may suggest that low-dose LSD preferentially modulates distributed feedback, arousal, and precision-weighting processes rather than early contextual gain control.

Future research on surround suppression should test higher LSD doses, earlier assessment time points, and psychedelic effects after 5-HT2A/1A or D2 antagonist pre-treatment or reversal. Research on low-dose LSD should probe the neurocognitive mechanisms underlying the subjective alterations observed here with paradigms indexing more distributed neurocognitive mechanisms.

## Supplementary Material

Supplementary_materials_niag050

## Data Availability

The data underlying this article will be shared on reasonable request to the corresponding author.
