# Supplementary material for: Low-dose lysergic acid diethylamide alters subjective visual perception but not visual contrast surround suppression
Source: Neurosci Conscious. 2026 Aug 20;2026(1):niag050. doi: 10.1093/nc/niag050 (PMC13492661; doi:10.1093/nc/niag050)
Supplement: Supplementary file 1 [file supplementary_materials_niag050.docx]

Supplement:

Low-dose LSD alters subjective visual perception but not visual contrast surround suppression.

Lucca F. Jaeckel*^1^, Deborah Logvinski^1^, Mika Kanana^1^, Felix Müller^2,3^, Nicolai Rohner^1^, Anna M. Becker^2,3^, Matthias E. Liechti^3^, Philipp Sterzer^1^

^1^University of Basel, Division of Translational Psychiatry, Department of Psychiatry (UPK) and Department of Clinical Research, Switzerland; ^2^University of Basel, Clinical Research Unit for Substance-Based Therapy, Department of Psychiatry (UPK), Switzerland; ^3^University Hospital Basel and University of Basel, Division of Clinical Pharmacology and Toxicology, Department of Biomedicine and Department of Clinical Research, Switzerland

*Corresponding Author. Division of Translational Psychiatry, Universitäre Psychiatrische Kliniken (UPK), Wilhelm Klein-Strasse 27, 4002 Basel, Switzerland. Email: [lucca.jaeckel@unibas.ch](mailto:philipp.sterzer@upk.ch)

# Supplementary Methods

## Data Exclusion

As registered, we used a pre-defined inclusion range for the point of subjective equality (PSE) that was our main outcome of 0–0.5. The lower value is the lowest reasonable contrast value the participants may perceive the target to have. The upper value was inspired by previous research by Schallmo et al. (2015), who excluded participants that likely compared the reference to the surround annulus instead of the target. Specifically, they excluded participants if they showed a PSE of ≥ 0.6 on average across different surround conditions or if they did not show a PSE ≤ 0.4 in any of the surround conditions. Since our psi-adaptive staircase only showed reference contrasts up to 0.55, we opted to exclude sessions with PSE estimates > 0.5. Of the 92 sessions performed by participants who completed at least two sessions, one was excluded because the participant pressed the wrong buttons on one study day. Six were excluded, because model fitting failed, producing either an Inf, -Inf, or NaN result for the threshold parameter. Five were excluded for having a finite PSE < 0, and seven were excluded for a finite PSE > 0.5. This resulted in 73 remaining sessions. After excluding participants with only one remaining session, this left 68 datapoints from 24 participants for the analysis. Below, we show exemplary data from an excluded and an included participant. As can be seen, the choices of the excluded participant #6 do not seem to follow any system, indicating that they generally had trouble performing the task (Supplementary Figure 1A). In contrast, the included participant #29 shows a clear sigmoid stimulus-response relationship that is well captured by the logistic model (Supplementary Figure 1B). The highlighted participants represent the overall sample and the respective included/excluded subsamples well. Of note, we excluded 6/30 placebo sessions, 7/31 10µg sessions, and 10/31 20µg sessions. A Cochran’s Q indicated no significant difference in the rate of exclusions across the conditions (*Q*(2) = 5.25, *p* = 0.072).

## Bayesian Analysis

We fitted Bayesian linear mixed models using the brms package using weakly to moderately informative priors. Specifically, we compared the full model (1) with a reduced model (2):

(1) Contrast_dec ~ DrugCondition + StudyDay + DrugCondition:StudyDay + (1 | SubjectID)
(2) Contrast_dec ~ StudyDay + (1 | SubjectID)

We used treatment coding so that the intercept corresponded to placebo on day 1, making it straightforward to set a prior for this baseline value. Our priors were informed by our pilot study and the previous studies probing surround suppression after psychedelics (Aqil et al., 2025; Swanson et al., 2024). Namely, for the intercept we set a moderately informative normal prior centered at -0.1 with standard deviation (SD) of 0.05, reflecting the expectation from pilot data that surround suppression at baseline would fall near -0.1, while still allowing for plausible deviations within approximately ±0.1. For the regression coefficients, we assigned a weakly to moderately informative normal prior centered at 0 with SD of 0.1, reflecting the expectation that the effect of drug condition or order would likely lie in a range of ± 0.2 as previous studies have found enhanced surround suppression due to psilocybin of up to about 10% across different tasks (Aqil et al., 2025; Swanson et al., 2024). We kept this prior centered at 0 because it also informed the estimates of the effect of study day and interaction term, where we did not have an expectation about the direction but did not expect effects on a different scale than for drug condition. For SD of the random intercepts and residual error we assigned weakly regularizing student-t priors with 3 degrees of freedom (df), centered at 0, and with σ of 0.15 for the random intercepts SD and a σ of 0.05 for the residual error. Therein, the df and center correspond to the default used by brms and the σ is adjusted to fit our outcome scale better than the brms default of 10. Also, the random intercept SD prior specifies that we expect moderate inter-individual variability. Posterior predictive checks indicated that the priors were not overly restrictive, as simulated data qualitatively replicated the distribution of the observed contrast suppression scores.

**Supplementary Figure 1:**

*Exemplary psychometric curves*

*Note.* Psychometric curves for two exemplary participants. Participant 29 (A) was included, whereas participant 6 (B) was excluded because all of their sessions were outside the pre-defined inclusion range of the point of subjective equivalence (PSE). Dots show the percentage of choice reference at a reference contrast level. Green lines show fitted psychometric functions. Inset text displays the PSE estimate. Blue bars show the share of samples presented at the respective contrast levels.

**Supplementary Figure 2**

*Maximum- and Minimum-Imputed Data*

*Note.* Contrast decrement per drug condition for the imputed data. Boxplots display the median (horizontal line), interquartile range (IQR; box boundaries), and minimum/maximum values within 1.5 × IQR (whiskers). Dots represent individual participant values, with grey lines connecting repeated measures. Red diamonds indicate the mean of each condition.

**Supplementary Table 1**

*Linear Mixed Effects Model Fixed and Random Effects*

|  | **Contrast decrement** | | | | | | | |
| --- | --- | --- | --- | --- | --- | --- | --- | --- |
| *Predictors* | *Estimates* | *std. Error* | | *CI* | *Statistic* | *p* | *df* |  |
| (Intercept) | -0.15 | 0.02 | | -0.20 – -0.11 | -6.46 | **<.001** | 22.45 |  |
| DrugCondition1 | 0.00 | 0.01 | | -0.02 – 0.03 | 0.37 | .710 | 36.49 |  |
| DrugCondition2 | -0.01 | 0.01 | | -0.03 – 0.01 | -0.96 | .341 | 36.97 |  |
| StudyDay1 | 0.02 | 0.01 | | -0.00 – 0.04 | 1.66 | .105 | 36.84 |  |
| StudyDay2 | -0.01 | 0.01 | | -0.03 – 0.01 | -1.11 | .275 | 36.51 |  |
| DrugCondition1:StudyDay1 | 0.03 | 0.02 | | -0.01 – 0.07 | 1.47 | .149 | 44.94 |  |
| DrugCondition2:StudyDay1 | -0.04 | 0.02 | | -0.08 – 0.00 | -1.92 | .061 | 44.07 |  |
| DrugCondition1:StudyDay2 | -0.03 | 0.02 | | -0.08 – 0.01 | -1.67 | .101 | 44.90 |  |
| DrugCondition2:StudyDay2 | 0.03 | 0.02 | | -0.01 – 0.07 | 1.51 | .139 | 45.63 |  |
| *Random Effects* | | |  | | | | | |
| σ^2^ | 0.00 | | | | | | | |
| τ_00_ _SubjectID_ | 0.01 | | | | | | | |
| ICC | 0.78 | | | | | | | |
| N _SubjectID_ | 24 | |  | | | | | |
| Observations | 68 | | | | | | | |
| Marginal R^2^ / Conditional R^2^ | 0.053 / 0.792 | | | | | | | |

*Note*. Fixed and random effects table of the linear mixed model. Note that since the factor variables were sum-coded, the estimates of the fixed effects for DrugCondition and StudyDay do not translate directly to specific levels of the variables. Significant p-values < .05 in bold. CI = Confidence interval; ICC = intraclass correlation coefficient; R² = coefficient of determination.

**Supplementary Table 2**

*Surround Suppression Psychedelic-Naïve Participants LMM Fixed Effects*

|  | **Contrast decrement** | | | | | | |
| --- | --- | --- | --- | --- | --- | --- | --- |
| *Predictors* | *Estimates* | *std. Error* | | *CI* | *Statistic* | *p* | *df* |
| (Intercept) | -0.10 | 0.03 | | -0.16 – -0.03 | -3.43 | **0.007** | 8.99 |
| DrugCondition1 | -0.01 | 0.01 | | -0.04 – 0.02 | -0.77 | 0.454 | 14.99 |
| DrugCondition2 | -0.01 | 0.01 | | -0.04 – 0.02 | -0.62 | 0.543 | 15.10 |
| StudyDay1 | 0.03 | 0.01 | | 0.00 – 0.05 | 2.17 | **0.046** | 15.02 |
| StudyDay2 | -0.02 | 0.01 | | -0.04 – 0.01 | -1.55 | 0.141 | 15.06 |
| *Random Effects* | | |  | | | | |
| σ^2^ | 0.00 | | | | | | |
| τ_00_ _SubjectID_ | 0.01 | | | | | | |
| ICC | 0.80 | | | | | | |
| N _SubjectID_ | 10 | |  | | | | |
| Observations | 29 | | | | | | |
| Marginal R^2^ / Conditional R^2^ | 0.047 / 0.814 | | | | | | |

*Note*. Fixed and random effects table of the linear mixed model fit to the data of only the participants with no prior psychedelic drug exposure. Note that due to the small n, the interaction of DrugCondition and StudyDay could not be estimated and since the factor variables were sum-coded, the estimates of the fixed effects for DrugCondition and StudyDay do not translate directly to specific levels of the variables. Significant p-values < .05 in bold. CI = Confidence interval; ICC = intraclass correlation coefficient; R² = coefficient of determination.

**Supplementary Table 3**

*Results Type-III ANOVA Psychedelic-Naïve Participants*

|  | SS | MS | Numerator df | Denominator df | F | p |
| --- | --- | --- | --- | --- | --- | --- |
| Drug Condition | 0.004 | 0.002 | 2 | 15.06 | 1.16 | 0.34 |
| StudyDay | 0.01 | 0.005 | 2 | 15.06 | 2.75 | 0.096 |

*Note.* SS = Sum of Squares, MS = Mean Squares, DF = Degrees of Freedom. Denominator df were estimated using Satterthwaite’s method.

**Supplementary Table 4**

*Sensitivity Analysis: Minimum-Imputed Data LMM*

|  | **Contrast decrement** | | | | | | |
| --- | --- | --- | --- | --- | --- | --- | --- |
| *Predictors* | *Estimates* | *std. Error* | | *CI* | *Statistic* | *p* | *df* |
| (Intercept) | -0.11 | 0.04 | | -0w.19 – -0.03 | -2.73 | **0.008** | 60.93 |
| DrugCondition1 | -0.06 | 0.06 | | -0.18 – 0.06 | -0.95 | 0.344 | 53.79 |
| DrugCondition2 | -0.06 | 0.05 | | -0.17 – 0.05 | -1.10 | 0.276 | 53.19 |
| StudyDay1 | -0.07 | 0.05 | | -0.17 – 0.04 | -1.29 | 0.201 | 53.80 |
| StudyDay2 | -0.13 | 0.06 | | -0.26 – -0.01 | -2.13 | **0.038** | 52.89 |
| DrugCondition1:StudyDay1 | 0.07 | 0.09 | | -0.10 – 0.25 | 0.86 | 0.396 | 58.01 |
| DrugCondition2:StudyDay1 | 0.07 | 0.09 | | -0.11 – 0.25 | 0.78 | 0.437 | 57.59 |
| DrugCondition1:StudyDay2 | 0.13 | 0.09 | | -0.05 – 0.31 | 1.47 | 0.146 | 55.34 |
| DrugCondition2:StudyDay2 | 0.09 | 0.09 | | -0.09 – 0.27 | 1.05 | 0.299 | 57.23 |
| *Random Effects* | | |  | | | | |
| σ^2^ | 0.01 | | | | | | |
| τ_00_ _SubjectID_ | 0.01 | | | | | | |
| ICC | 0.55 | | | | | | |
| N _SubjectID_ | 24 | |  | | | | |
| Observations | 72 | | | | | | |
| Marginal R^2^ / Conditional R^2^ | 0.054 / 0.570 | | | | | | |

*Note*. Fixed and random effects table of the linear mixed model fit to the minimum-imputed data. Note that since the factor variables were sum-coded, the estimates of the fixed effects for DrugCondition and StudyDay do not translate directly to specific levels of the variables. Significant p-values < .05 in bold. CI = Confidence interval; ICC = intraclass correlation coefficient; R² = coefficient of determination.

**Supplementary Table 5**

*Results Type-III ANOVA Minimum-Imputed Data*

|  | SS | MS | Numerator df | Denominator df | F | p |
| --- | --- | --- | --- | --- | --- | --- |
| Drug Condition | 0.004 | 0.002 | 2 | 40.94 | 0.22 | .801 |
| Study Day | 0.036 | 0.018 | 2 | 40.94 | 2.04 | .144 |
| Drug Condition: Study Day | 0.020 | 0.005 | 4 | 55.33 | 0.58 | .676 |

*Note.* SS = Sum of Squares, MS = Mean Squares, DF = Degrees of Freedom. Denominator df were estimated using Satterthwaite’s method.

**Supplementary Table 6**

*Sensitivity Analysis: Maximum-Imputed Data LMM*

|  | **Contrast decrement** | | | | | | |
| --- | --- | --- | --- | --- | --- | --- | --- |
| *Predictors* | *Estimates* | *std. Error* | | *CI* | *Statistic* | *p* | *df* |
| (Intercept) | -0.09 | 0.03 | | -0.16 – -0.02 | -2.57 | **0.013** | 59.89 |
| DrugCondition1 | -0.11 | 0.05 | | -0.21 – -0.01 | -2.20 | **0.032** | 52.39 |
| DrugCondition2 | -0.02 | 0.05 | | -0.11 – 0.07 | -0.50 | 0.618 | 51.77 |
| StudyDay1 | -0.12 | 0.04 | | -0.21 – -0.04 | -2.81 | **0.007** | 52.37 |
| StudyDay2 | -0.04 | 0.05 | | -0.14 – 0.07 | -0.70 | 0.490 | 51.52 |
| DrugCondition1:StudyDay1 | 0.19 | 0.07 | | 0.05 – 0.34 | 2.62 | **0.011** | 56.15 |
| DrugCondition2:StudyDay1 | 0.08 | 0.07 | | -0.07 – 0.23 | 1.05 | 0.299 | 55.76 |
| DrugCondition1:StudyDay2 | 0.06 | 0.08 | | -0.09 – 0.22 | 0.82 | 0.418 | 53.71 |
| DrugCondition2:StudyDay2 | 0.03 | 0.08 | | -0.13 – 0.18 | 0.36 | 0.724 | 55.37 |
| *Random Effects* | | |  | | | | |
| σ^2^ | 0.01 | | | | | | |
| τ_00_ _SubjectID_ | 0.01 | | | | | | |
| ICC | 0.60 | | | | | | |
| N _SubjectID_ | 24 | |  | | | | |
| Observations | 72 | | | | | | |
| Marginal R^2^ / Conditional R^2^ | 0.098 / 0.637 | | | | | | |

*Note*. Fixed and random effects table of the linear mixed model fit to the maximum-imputed data. Note that since the factor variables were sum-coded, the estimates of the fixed effects for DrugCondition and StudyDay do not translate directly to specific levels of the variables. Significant p-values < .05 in bold. CI = Confidence Interval; ICC = Intraclass Correlation Coefficient; R² = Coefficient of Determination.

**Supplementary Table 7**

|  | SS | MS | Numerator df | Denominator df | F | p |
| --- | --- | --- | --- | --- | --- | --- |
| Drug Condition | 0.015 | 0.008 | 2 | 41.12 | 1.25 | .299 |
| Study Day | 0.014 | 0.007 | 2 | 41.12 | 1.17 | .321 |
| Drug Condition: Study Day | 0.046 | 0.012 | 4 | 53.72 | 1.88 | .127 |

*Results Type-III ANOVA Maximum-Imputed Data*

*Note.* SS = Sum of Squares, MS = Mean Squares, DF = Degrees of Freedom. Denominator df were estimated using Satterthwaite’s method.

**Supplementary Table 8**

*Choice Reaction Times Model*

|  | **Average Reaction Time** | | | | | | |
| --- | --- | --- | --- | --- | --- | --- | --- |
| *Predictors* | *Estimates* | *std. Error* | | *CI* | *Statistic* | *p* | *df* |
| (Intercept) | 0.39 | 0.07 | | 0.26 – 0.52 | 5.94 | **<0.001** | 50.85 |
| DrugCondition1 | 0.08 | 0.08 | | -0.10 – 0.25 | 0.89 | 0.376 | 44.82 |
| DrugCondition2 | 0.08 | 0.08 | | -0.08 – 0.24 | 1.00 | 0.325 | 42.82 |
| StudyDay1 | -0.04 | 0.08 | | -0.19 – 0.11 | -0.56 | 0.581 | 44.68 |
| StudyDay2 | -0.13 | 0.09 | | -0.31 – 0.06 | -1.36 | 0.180 | 42.08 |
| DrugCondition1:StudyDay1 | 0.01 | 0.13 | | -0.25 – 0.27 | 0.08 | 0.939 | 47.90 |
| DrugCondition2:StudyDay1 | -0.02 | 0.13 | | -0.28 – 0.23 | -0.18 | 0.859 | 45.35 |
| DrugCondition1:StudyDay2 | 0.00 | 0.13 | | -0.27 – 0.27 | 0.00 | 0.997 | 44.11 |
| DrugCondition2:StudyDay2 | 0.05 | 0.14 | | -0.22 – 0.33 | 0.40 | 0.693 | 44.19 |
| *Random Effects* | | |  | | | | |
| σ^2^ | 0.02 | | | | | | |
| τ_00_ _SubjectID_ | 0.05 | | | | | | |
| ICC | 0.76 | | | | | | |
| N _SubjectID_ | 24 | |  | | | | |
| Observations | 68 | | | | | | |
| Marginal R^2^ / Conditional R^2^ | 0.044 / 0.768 | | | | | | |

*Note*. Fixed and random effects table of the linear mixed model fit to average reaction times with the model structure mean_rt ~ DrugCondition * StudyDay + (1 | proband). Significant p-values < .05 in bold. CI = Confidence Interval; ICC = Intraclass Correlation Coefficient; R² = Coefficient of Determination.

**Supplementary Table 9**

|  | SS | MS | Numerator df | Denominator df | F | p |
| --- | --- | --- | --- | --- | --- | --- |
| Drug Condition | 0.09 | 0.05 | 2 | 37.30 | 2.87 | 0.069 |
| Study Day | 0.11 | 0.05 | 2 | 37.18 | 3.46 | **0.041** |
| Drug Condition: Study Day | 0.01 | 0.00 | 4 | 44.32 | 0.14 | 0.964 |

*Results Type-III ANOVA Average Reaction Times*

*Note.* SS = Sum of Squares, MS = Mean Squares, DF = Degrees of Freedom. Denominator df were estimated using Satterthwaite’s method.

**Supplementary Table 10**

*All VAS Items Average Marginal Effect of Dose Escalation*

|  | 10µg - Placebo | | 20µg - Placebo | | 20µg - 10µg | |
| --- | --- | --- | --- | --- | --- | --- |
| VAS Item | Estimate | 95% CI | Estimate | 95% CI | Estimate | 95% CI |
| VAS-1 “Any drug effect” | **4.933** | **[0.880, 9.412]** | **17.124** | **[11.418, 23.185]** | **12.150** | **[5.919, 18.413]** |
| VAS-2 “Good drug effect” | 4.268 | [-0.056, 8.785] | **11.946** | **[6.867, 17.553]** | **7.697** | **[2.052, 13.677]** |
| VAS-3 “Bad drug effect” | 0.746 | [-0.656, 2.386] | **2.262** | **[0.643, 4.979]** | 1.523 | [-0.218, 3.972] |
| VAS-4 “Feeling high” | 2.221 | [-0.425, 5.662] | **7.190** | **[3.558, 12.204]** | **4.910** | **[0.538, 10.082]** |
| VAS-5 “Anxious” | -0.130 | [-1.338, 0.559] | 1.063 | [-0.021, 4.635] | **1.223** | **[0.156, 5.007]** |
| VAS-6 “Nauseous” | -0.257 | [-2.122, 1.373] | **2.184** | **[0.323, 4.970]** | **2.465** | **[0.637, 5.381]** |
| VAS-7 “Depressed” | -0.083 | [-2.002, 1.601] | 1.512 | [-0.267, 4.306] | 1.597 | [-0.292, 4.763] |
| VAS-8 “Altered Visual Perception” | 2.574 | [-0.112, 5.552] | **7.510** | **[4.233, 11.536]** | **4.956** | **[1.272, 8.984]** |
| VAS-9 “Altered auditory perception” | 0.351 | [-2.195, 2.769] | **2.901** | **[0.203, 6.454]** | 2.547 | [-0.408, 6.438] |
| VAS-10 “Auditory-visual synesthesia” | 0.970 | [-0.619, 3.306] | 1.203 | [-0.543, 4.676] | 0.240 | [-2.066, 3.441] |
| VAS-11 “Altered perception of time” | **3.126** | **[0.926, 5.843]** | **8.930** | **[5.792, 12.998]** | **5.786** | **[2.269, 9.783]** |
| VAS-12 “Loosening of self-world boundaries” | 1.090 | [-1.053, 3.575] | **4.423** | **[1.721, 8.421]** | **3.330** | **[0.266, 7.417]** |
| VAS-13 “Gaining new insights” | 0.835 | [-1.074, 3.070] | 1.199 | [-0.654, 3.831] | 0.362 | [-1.897, 3.057] |
| VAS-14 “More/less talkative” | Ratio of positive/negative values not statistically significantly different from 0.5 | | | | | |
| VAS-15 “More/less open” | Ratio of positive/negative values not statistically significantly different from 0.5 | | | | | |
| VAS-16 “More/less trusting” | 0.142 | [-1.780, 2.578] | 1.199 | [-0.885, 4.329] | 1.024 | [-1.386, 4.214] |
| VAS-17 “More/fewer personal thoughts” | Ratio of positive/negative values not statistically significantly different from 0.5 | | | | | |
| VAS-18 “Blurrier/sharper visual perception” | **-3.665** | **[-6.914, -0.877]** | **-7.222** | **[-11.338, -3.724]** | -3.555 | [-7.705, 0.716] |
| VAS-19 “Less/more saturated visual perception” | -0.919 | [-3.100, 0.364] | **2.628** | **[0.381, 6.132]** | **3.602** | **[1.384, 7.589]** |
| VAS-20 “Darker/brighter visual perception” | 0.590 | [-1.442, 3.382] | 2.212 | [-0.005, 5.336] | 1.569 | [-1.331, 4.847] |
| VAS-21 “More static/dynamical visual perception | 1.250 | [-0.180, 3.358] | **3.655** | **[1.862, 6.970]** | **2.388** | **[0.454, 5.296]** |

*Note.* Average marginal effects represent the expected change in the outcome for the specified contrast, averaged across all subjects (with random intercepts integrated out) and over the distribution of covariates. For instance, the upper left value gives the average increase in ratings of any drug effect after administration of 10 µg compared to placebo*.* Estimates and 95% credible intervals (CI) are in bold where the CI does not include zero and hence, we interpret the estimated average marginal effect to be credibly different from 0. VAS = Visual Analog Scale.

**Supplementary Table 11**

*11ASC Average Marginal Effect of Dose Escalation*

|  | 10µg - Placebo | | 20µg - Placebo | | 20µg - 10µg | |  |
| --- | --- | --- | --- | --- | --- | --- | --- |
| 11ASC Factor | Estimate | 95% CI | Estimate | 95% CI | Estimate | 95% CI | |
| Experience of Unity | 2.732 | [-0.266, 5.929] | 1.366 | [-1.324, 4.169] | -1.378 | [-4.557, 1.696] | |
| Spiritual Experience | -0.699 | [-3.409, 0.786] | 0.864 | [-1.292, 3.994] | 1.583 | [-0.220, 5.486] | |
| Blissful State | 0.376 | [-1.451, 2.411] | **3.511** | **[0.586, 8.115]** | 3.148 | [-0.046, 7.595] | |
| Insightfulness | 0.921 | [-1.010, 3.440] | **3.976** | **[1.119, 8.314]** | 3.023 | [-0.082, 7.204] | |
| Disembodiment | **1.909** | **[0.311, 4.099]** | **8.583** | **[5.171, 13.344]** | **6.651** | **[3.047, 11.155]** | |
| Impaired control and Cognition | 0.500 | [-0.871, 2.038] | **2.705** | **[0.898, 5.884]** | **2.213** | **[0.324, 5.253]** | |
| Anxiety | 1.784 | [-0.304, 4.189] | 1.872 | [-0.006, 4.125] | 0.103 | [-2.387, 2.469] | |
| Complex Imagery | 0.752 | [-0.702, 3.107] | 0.745 | [-0.931, 3.511] | -0.033 | [-2.236, 2.545] | |
| Elementary Imagery | 1.685 | [-0.255, 4.668] | **3.389** | **[0.512, 8.514]** | 1.666 | [-2.204, 6.907] | |
| Audio-Visual Synesthesia | 2.680 | [-0.483, 6.042] | **6.325** | **[2.774, 10.359]** | 3.615 | [-0.236, 7.909] | |
| Changed Meaning of Percepts | 0.331 | [-1.390, 2.024] | **2.887** | **[0.421, 6.669]** | **2.560** | **[0.123, 6.305]** | |

*Note.* Average marginal effects represent the expected change in the rating of the items of the factor for the specified contrast, averaged across all subjects (with random intercepts integrated out) and over the distribution of covariates. For instance, the upper left value gives the average increase in ratings of the items forming the “Experience of unity” factor after administration of 10 µg compared to placebo*.* Estimates and 95% credible intervals (CI) are in bold where the CI does not include zero and hence, we interpret the estimated average marginal effect to be credibly different from 0. 5D-ASC = 5-Dimensional Altered State of Consciousness Rating Scale; 11ASC = 11-factor scoring variant of the 5D-ASC.

**Supplementary Table 12**

*Subjective Effects Average Marginal Effect of Study Day*

|  | **Study Day 2 - Study Day 1** | | **Study Day 3 - Study Day 1** | | **Study Day 3 - Study Day 2** | |
| --- | --- | --- | --- | --- | --- | --- |
| **VAS Item / Subscale** | **Estimate** | **95% CI** | **Estimate** | **95% CI** | **Estimate** | **95% CI** |
| Selected VAS Items | | | | | | |
| VAS-1: “Any drug effect” | -1.338 | [-7.165, 4.143] | -3.350 | [-8.886, 2.047] | -2.007 | [-7.087, 3.351] |
| VAS-2: “Good drug effect” | -4.377 | [-10.245, 1.155] | -5.308 | [-10.742, 0.019] | -0.934 | [-5.723, 3.951] |
| VAS-3: “Bad drug effect” | -0.385 | [-2.471, 1.404] | -0.889 | [-3.004, 0.732] | -0.491 | [-2.481, 1.248] |
| VAS-8 “Altered visual perception” | -3.449 | [-7.293, 0.082] | **-5.060** | **[-8.765, -1.694]** | -1.605 | [-4.615, 1.239] |
| VAS-18: “Blurrier vs. sharper” | 2.358 | [-1.547, 6.486] | **4.505** | **[0.887, 8.588]** | 2.188 | [-1.057, 5.485] |
| VAS-19: “Less vs. more saturated” | -0.138 | [-2.759, 2.335] | -0.681 | [-3.152, 2.055] | -0.502 | [-3.292, 2.338] |
| VAS-20: “Darker vs. brighter” | -0.411 | [-3.645, 2.181] | 0.115 | [-3.037, 3.077] | 0.523 | [-1.764, 3.271] |
| VAS-21: “Static vs. dynamical” | -1.163 | [-3.673, 0.960] | -1.630 | [-4.071, 0.296] | -0.475 | [-2.353, 1.344] |
| 5D-ASC Dimensions | | | | | | |
| Oceanic Boundlessness | -1.473 | [-4.557, 0.861] | **-2.462** | **[-5.763, -0.361]** | -1.002 | [-3.132, 0.701] |
| Anxious Ego-Dissolution | 0.124 | [-1.993, 2.188] | -0.711 | [-2.819, 1.047] | -0.833 | [-2.777, 0.871] |
| Visionary Restructuralization | -1.285 | [-4.849, 1.909] | **-2.787** | **[-6.414, -0.067]** | -1.524 | [-4.376, 0.813] |
| Auditory Alterations | -0.045 | [-1.916, 1.565] | 0.297 | [-1.431, 2.149] | 0.364 | [-1.229, 2.134] |
| Vigilance Reduction | -4.327 | [-8.628, 0.040] | **-5.285** | **[-9.512, -1.014]** | -0.946 | [-4.502, 2.451] |

*Note.* Average marginal effects represent the expected increase or decrease in the rating of the item/dimension for the specified step in time, averaged over the sample, accounting for covariates and subject-level random intercepts. For instance, the upper left value gives the average change in ratings of “Any drug effect” on the second compared to the first study day*.* Estimates and 95% credible intervals (CI) are in bold where the CI does not include zero and hence, we interpret the estimated average marginal effect to be credibly different from 0. VAS = Visual Analog Scale.

**Supplementary Table 13**

*Selected VAS Items Conditional Average Treatment Effect Difference by Study Day*

|  | Study Day 2 - Study Day 1 | | Study Day 3 - Study Day 1 | | Study Day 3 - Study Day 2 | |
| --- | --- | --- | --- | --- | --- | --- |
| VAS Item | Estimate | CI | Estimate | CI | Estimate | CI |
| Placebo | | | | | | |
| VAS-1: “Any drug effect” | -4.846 | [-11.330, 0.316] | -1.193 | [-8.190, 5.509] | 3.632 | [-1.244, 9.653] |
| VAS-2: “Good drug effect” | -3.336 | [-10.165, 1.797] | -0.387 | [-7.608, 6.405] | 2.920 | [-2.063, 9.490] |
| VAS-3: “Bad drug effect” | -0.297 | [-3.616, 2.315] | -0.839 | [-4.347, 0.620] | -0.505 | [-3.233, 0.889] |
| VAS-8 “Altered visual perception” | -0.308 | [-4.919, 4.703] | -0.906 | [-5.089, 2.505] | -0.533 | [-5.451, 3.002] |
| VAS-18: “Blurrier vs. sharper” | -0.817 | [-5.166, 2.763] | -2.954 | [-8.350, 1.194] | -2.106 | [-7.668, 2.765] |
| VAS-19: “Less vs. more saturated” | -1.334 | [-6.305, 1.389] | -0.212 | [-4.782, 3.819] | 1.114 | [-1.695, 5.514] |
| VAS-20: “Darker vs. brighter” | -1.211 | [-4.487, 0.446] | 1.529 | [-2.001, 6.788] | **2.785** | **[0.382, 8.201]** |
| VAS-21: “Static vs. dynamical” | **-1.493** | **[-4.992, -0.191]** | -0.748 | [-4.089, 1.467] | 0.677 | [-0.183, 2.914] |
| 10 µg | | | | | | |
| VAS-1: “Any drug effect” | **-9.740** | **[-19.719, -1.203]** | -6.233 | [-16.558, 3.190] | 3.577 | [-3.388, 10.806] |
| VAS-2: “Good drug effect” | **-11.518** | **[-22.585, -2.127]** | -8.467 | [-19.680, 1.774] | 3.022 | [-4.008, 10.629] |
| VAS-3: “Bad drug effect” | **-3.635** | **[-8.604, -1.453]** | -2.674 | [-7.324, 0.545] | **0.853** | **[0.062, 4.003]** |
| VAS-8 “Altered visual perception” | **-7.041** | **[-14.848, -0.553]** | **-7.927** | **[-15.486, -2.243]** | -0.868 | [-5.573, 2.934] |
| VAS-18: “Blurrier vs. sharper” | **8.936** | **[0.371, 18.677]** | **10.503** | **[3.198, 20.171]** | 1.503 | [-3.291, 7.479] |
| VAS-19: “Less vs. more saturated” | -0.589 | [-4.037, 0.057] | 0.177 | [-3.050, 2.916] | **0.803** | **[0.022, 3.647]** |
| VAS-20: “Darker vs. brighter” | -0.196 | [-7.822, 4.484] | -0.326 | [-7.726, 3.585] | -0.133 | [-4.366, 3.411] |
| VAS-21: “Static vs. dynamical” | -2.870 | [-8.076, 0.350] | -1.701 | [-6.689, 1.571] | 1.163 | [-1.417, 4.140] |
| 20 µg | | | | | | |
| VAS-1: “Any drug effect” | 10.847 | [-2.981, 24.221] | -2.347 | [-14.797, 9.542] | -13.188 | [-26.432, 0.083] |
| VAS-2: “Good drug effect” | 1.868 | [-12.094, 15.215] | -6.680 | [-19.537, 5.260] | -8.602 | [-20.463, 2.887] |
| VAS-3: “Bad drug effect” | 2.847 | [-1.083, 8.863] | 1.038 | [-2.873, 5.709] | -1.835 | [-7.748, 2.973] |
| VAS-8 “Altered visual perception” | -2.695 | [-12.220, 6.003] | -5.906 | [-15.260, 2.181] | -3.215 | [-10.789, 3.989] |
| VAS-18: “Blurrier vs. sharper” | -1.022 | [-11.865, 10.118] | 5.852 | [-2.427, 16.063] | 6.929 | [-1.433, 16.348] |
| VAS-19: “Less vs. more saturated” | 1.770 | [-5.268, 10.205] | -1.922 | [-8.635, 5.996] | -3.745 | [-12.483, 4.818] |
| VAS-20: “Darker vs. brighter” | 0.591 | [-6.701, 6.754] | -0.678 | [-8.068, 6.318] | -1.212 | [-7.383, 5.340] |
| VAS-21: “Static vs. dynamical” | 1.291 | [-4.927, 7.332] | -2.083 | [-7.806, 2.433] | -3.393 | [-9.087, 1.479] |

*Note*. Conditional treatment effects represent the expected change in the outcome for a given contrast of study days, estimated separately within each treatment condition. These effects account for subject-level random intercepts but are not averaged across treatment conditions. For instance, the upper left value gives the average change in ratings of “Any drug effect” on the second compared to the first study day, conditional on participants having received placebo. Estimates and 95% credible intervals (CI) are in bold where the CI does not include zero and hence, we interpret the estimated conditional average treatment effect difference to be credibly different from 0. VAS = Visual Analog Scale.

**Supplementary Table 14**

*5D-ASC Conditional Average Treatment Effect Difference by Study Day*

|  | Study Day 2 - Study Day 1 | | Study Day 3 - Study Day 1 | | Study Day 3 - Study Day 2 | |
| --- | --- | --- | --- | --- | --- | --- |
| 5D-ASC Subscale | Estimate | 95% CI | Estimate | 95% CI | Estimate | 95% CI |
| Placebo | | | | | | |
| Oceanic Boundlessness | -0.732 | [-4.057, 2.829] | -1.025 | [-4.227, 1.389] | -0.275 | [-3.850, 2.153] |
| Anxious Ego-Dissolution | 1.448 | [-1.318, 5.443] | 0.490 | [-1.976, 3.621] | -0.973 | [-4.990, 2.351] |
| Visionary Restructuralization | 0.961 | [-2.485, 6.053] | -1.090 | [-4.082, 0.801] | -2.104 | [-7.230, 0.312] |
| Auditory Alterations | 1.347 | [-0.355, 4.873] | 1.204 | [-0.435, 4.591] | -0.111 | [-3.418, 3.095] |
| Vigilance Reduction | 1.686 | [-4.898, 8.530] | -2.956 | [-8.974, 2.599] | -4.640 | [-11.226, 1.379] |
| 10 µg | | | | | | |
| Oceanic Boundlessness | -4.297 | [-11.692, 0.090] | **-5.203** | **[-12.732, -1.281]** | -0.807 | [-3.766, 1.349] |
| Anxious Ego-Dissolution | -2.089 | [-7.406, 0.680] | -1.487 | [-6.690, 1.786] | 0.590 | [-1.481, 3.367] |
| Visionary Restructuralization | -3.207 | [-9.912, 2.377] | **-4.689** | **[-11.244, -0.801]** | -1.438 | [-6.046, 1.520] |
| Auditory Alterations | -2.080 | [-7.734, 0.694] | -1.721 | [-7.135, 1.196] | 0.352 | [-1.685, 2.802] |
| Vigilance Reduction | **-10.392** | **[-19.802, -1.322]** | **-8.711** | **[-17.985, -0.361]** | 1.627 | [-5.247, 8.176] |
| 20 µg | | | | | | |
| Oceanic Boundlessness | 0.831 | [-5.307, 6.246] | -0.901 | [-6.924, 3.770] | -1.723 | [-6.952, 2.769] |
| Anxious Ego-Dissolution | 1.166 | [-3.118, 5.688] | -1.023 | [-5.198, 2.747] | -2.180 | [-6.823, 1.740] |
| Visionary Restructuralization | -1.513 | [-11.592, 6.244] | -2.121 | [-12.012, 5.237] | -0.513 | [-7.702, 5.932] |
| Auditory Alterations | 0.572 | [-2.505, 4.203] | 1.368 | [-1.931, 5.972] | 0.784 | [-2.943, 5.344] |
| Vigilance Reduction | -4.190 | [-13.963, 4.732] | -3.901 | [-13.453, 4.830] | 0.226 | [-7.218, 7.811] |

*Note*. Conditional average treatment effect differences represent the expected change in ratings of a 5D-ASC dimension between study days, separately for each treatment condition, averaged over the sample while accounting for subject-level random intercepts. For instance, the upper left value gives the average change in ratings of the items forming the “Experience of unity” factor on the second compared to the first study day, conditional on participants having received placebo. Estimates and 95% credible intervals (CI) are in bold where the CI does not include zero and hence, we interpret the estimated conditional average treatment effect difference to be credibly different from 0. 5D-ASC = Five-dimensional altered state of consciousness rating scale.

# References

Aqil, M., De Hollander, G., Vreugdenhil, N., Knapen, T., & Dumoulin, S. O. (2025). *Psilocybin alters visual contextual computations*. https://doi.org/10.1101/2025.02.06.636848

Schallmo, M.-P., Sponheim, S. R., & Olman, C. A. (2015). Reduced contextual effects on visual contrast perception in schizophrenia and bipolar affective disorder. *Psychological Medicine*, *45*(16), 3527–3537. https://doi.org/10.1017/S0033291715001439

Swanson, L. R., Jungers, S., Varghese, R., Cullen, K. R., Evans, M. D., Nielson, J. L., & Schallmo, M.-P. (2024). Enhanced visual contrast suppression during peak psilocybin effects: Psychophysical results from a pilot randomized controlled trial. *Journal of Vision*, *24*(12), 5. https://doi.org/10.1167/jov.24.12.5
